# Elucidating the gut microbiota-driven crosstalk: mechanistic interplay of lobetyolin in coordinating cholesterol homeostasis and anti-inflammatory pathways in hyperlipidemic mice models

**DOI:** 10.3389/fmicb.2025.1625211

**Published:** 2025-08-19

**Authors:** Guofeng Duan, Yuning Zhang, Siyuan Liu, Siqi Wang, Jinjia Liu, Lijuan Li, Lina Lai

**Affiliations:** ^1^School of Pharmacy, Changzhi Medical College, Changzhi, Shanxi, China; ^2^Shanxi Provincial Department-Municipal Key Laboratory Cultivation Base for Quality Enhancement and Utilization of Shangdang Chinese Medicinal Materials, Changzhi Medical College, Changzhi, Shanxi, China; ^3^School of Nursing, Changzhi Medical College, Changzhi, Shanxi, China

**Keywords:** enteritis, hyperlipidemia, gut microbiota, lobetyolin (LBT), Triton WR-1339

## Abstract

**Background:**

Hyperlipidemia is a prevalent metabolic disorder closely associated with gut microbiota imbalance. In recent years, traditional Chinese medicine has demonstrated distinct advantages in the regulation of the gut microbiota and enhancement of metabolic health. This study aimed to elucidate the molecular processes by which lobetyolin modifies the gut microbiota to improve intestinal inflammation and lipid metabolism in hyperlipidemic mice.

**Methods:**

Forty female KM mice were randomly allocated to four groups: control, model, LBT1, and LBT2. Mice in the LBT1 and LBT2 groups received intraperitoneal injections of the corresponding concentrations of LBT for ten consecutive days, whereas mice in the control and model groups received intraperitoneal injections of physiological saline. Beginning on the eighth day, mice in the model, LBT1, and LBT2 groups received subcutaneous injections of Triton WR-1339 for three consecutive days, whereas those in the control group received subcutaneous injections of physiological saline concurrently. On the eleventh day of the experiment, serum, liver, colon, and fecal samples were collected from all mice. This study aimed to measure lipid metabolism in mouse serum and liver, assess the inflammatory status of the mouse colon, and evaluate changes in the gut microbiota.

**Results:**

Lobetyolin significantly reduced the levels of triglycerides (TG), low-density lipoprotein cholesterol (LDL-C), very low-density lipoprotein cholesterol (VLDL-C), and total cholesterol (T-CHO) in the serum of hyperlipidemic mice. Concurrently, it elevated the levels of high-density lipoprotein cholesterol (HDL-C). The mechanism involves the reduction of endogenous cholesterol production and promotion of reverse cholesterol transport. LBT can also alleviate inflammatory responses by inhibiting the TLR4/NF-κB signaling pathway. In addition, it can regulate the balance of Th1 and Th2 immunity and enhance the immune capacity of the colon mucosa. According to the results of 16S rRNA sequencing, LBT increased the abundance of beneficial gut microbiota, such as *Akkermansia*, *Dubosiella*, and *Lactobacillus*, which were positively correlated with HDL-C, IL-10, IL-4, and SIgA but negatively correlated with T-CHO, TG, LDL-C, VLDL-C, IL-6, IFN-γ, and TNF-α.

**Conclusion:**

Our findings emphasize that lobetyolin exerts lipid-lowering and anti-inflammatory effects by regulating the ecological structure of the gut microbiota.

## 1 Introduction

Hyperlipidemia is characterized by elevated lipid concentrations, including cholesterol and triglyceride levels, in the bloodstream (Chevenon et al., 2022; Akeel, 2023). This illness is prevalent worldwide and affects both developed and developing countries. As lifestyle has progressed, the incidence of this disease has consistently increased. Research has demonstrated that Cardiovascular diseases rank among the foremost causes of mortality globally, with hyperlipidemia identified as a substantial risk factor (Qiu et al., 2021; Tien et al., 2021). Consequently, it has become a significant public health concern (Alloubani et al., 2021). Statins are the primary drugs used to treat hyperlipidemia. Nonetheless, certain patients may experience adverse effects, such as myalgia and hepatic impairment, necessitating consistent surveillance (Ruscica et al., 2022). Conversely, traditional Chinese medicine provides distinct benefits in the modulation of blood lipid levels (Jin and Wang, 2024).

*Codonopsis pilosula*, also known as Huang Shen or Lu Dang Shen, is a Chinese herbal remedy belonging to the Araliaceae family. The roots are used for medical purposes and are acknowledged for their diverse therapeutic properties, including tonifying qi, nourishing the blood, and moistening the lungs. These characteristics have resulted in their extensive use in both clinical and conventional settings. The Chinese Pharmacopoeia states that *Codonopsis pilosula* has a sweet and neutral flavor, efficiently replaces middle qi shortage, and moistens the lungs to relieve coughing. The principal components of this herb are glycosides, polysaccharides, and flavonoids. Current pharmacological research suggests that *Codonopsis pilosula* may reduce fatigue, bolster immunity, and exert antioxidant effects (Bailly, 2021). Its extracts also aid in lipid processing, which in turn reduces cholesterol and triglyceride levels, making it an important tool for treating metabolic diseases and repairing damaged pancreas and liver (Guo et al., 2024; Cha et al., 2012; Mao et al., 2022). Nonetheless, few studies have examined how different parts of *Codonopsis pilosula* extract affect hyperlipidemia; hence, future studies should focus on identifying the specific parts of this plant and how they work.

Lobetyolin, an essential active constituent of *Codonopsis pilosula*, possesses a polyacetylene core structure defined by a carbon atom chain interconnected by alternating double and single bonds. Its unique structure confers significant reactivity, facilitating interactions with biological systems. Its molecular structure comprises one or more sugar units, and glycosylation markedly improves its solubility and biological activity (Chu et al., 2024). Previous studies have demonstrated that lobetyolin shows favorable metabolic stability in liver microsomes (Tadege et al., 2022; Xie et al., 2023) and maintains excellent chemical stability across diverse pH conditions, thereby preserving its activity during metabolic processes in organisms (Liao et al., 2020) and exhibiting a favorable safety profile (Bailly, 2021). Furthermore, it exhibits a broad range of biological and pharmacological activities, including antitumor (Bailly, 2021; Cheng et al., 2023), neuroprotective (Wang et al., 2020), immunomodulatory (Liu et al., 2024; Chen et al., 2024), antiviral (Ni et al., 2022), cardioprotective (Liu et al., 2018), and antioxidant (Ni et al., 2023) activities. How lobetyolin, mediated by the gut microbiota, affects colonic inflammation and lipid metabolism in mice with metabolic-associated fatty liver disease (been). This study employed female Kunming mice and Triton WR-1339 as internal controls to investigate the role of lobetyolin in regulating lipid metabolism and the NF-κB signaling pathway in DSS-induced colitis. The impact of lobetyolin on gut microbiota was investigated to provide guidance for its development and application and lay the foundation for future functional research utilizing lobetyolin.

## 2 Materials and methods

### 2.1 The main chemical and biological reagents

Triton WR-1339 was sourced from Sigma-Aldrich in St. Louis, Missouri, USA. Furthermore, Lobetyolin originated from the same provider. Wuhan Servicebio Technology Co., Ltd., supplied the TRIzol reagent, PrimeScript RT reagent, and quantitative real-time polymerase chain reaction (QPCR) kits. We procured kits for AST, TC, TG, LDL-C, VLDL-C, and HDL-C from Nanjing Jiancheng Biotechnology Co., Ltd., Assay kits for IL-6, TNF-α, INF-γ, IL-4, IL-10, and SIgA were procured from AMOYLUNCHANGSHUOBIOTECH Co., All chemicals and solvents used in this study were of the highest commercially available quality.

### 2.2 Animals and treatments

**FIGURE 1:**
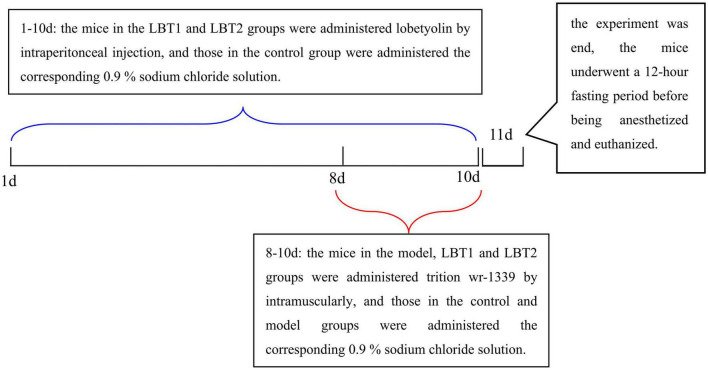
Experimental animal processing diagram.

Forty female Kunming mice were purchased from SpePharm Biotechnology Co., Ltd., (Beijing, China), and weighed 18–22 g. Prior to the start of the experiment, the mice were allowed to acclimatize to the laboratory. The individuals were housed in a controlled environment with a reversed 12-hour light/dark cycle, a temperature of 22 ± 1 °C, and a relative humidity of 50 ± 5 %. The mice had unrestricted access to food and water *ad libitum*. All animal procedures adhered to the guidelines established by the Animal Ethics Committee of Changzhi Medical College (Reference Number: DW2024070). Mice were randomly divided into four groups (control, model, LBT1, and LBT2; *n* = 10 per group) following the protocol described by Xiao et al. (2023), as illustrated in Figure 1. After a 12-hour fast, the animals received daily intraperitoneal injections for 10 consecutive days: the LBT1 and LBT2 groups were administered LBT at 10 and 50 mg kg^–1^ body weight, respectively (Ni et al., 2023), whereas the control and model groups received an equivalent volume of 0.9 % saline. Triton WR-1339 was freshly prepared in physiological saline at a concentration of 30 mg mL^–1^. On days 8–10 of treatment, all groups except the control group were injected intramuscularly with Triton WR-1339 at 480 mg kg^–1^ to induce acute hyperlipidemia. On day 11, the mice were fasted for 12 h, anesthetized, and euthanized.

### 2.3 Sample collecting and treatment

At the end of the experiment, the mice were fasted for 12 h. Fecal pellets were then gently collected by squeezing the perianal area near the tail base, immediately transferred into sterile centrifuge tubes, and stored at −80°C. All mice were subsequently anesthetized and euthanized, and blood was drawn from the orbital sinus and allowed to clot at 4°C. After centrifugation at 3000 rpm for 10 min, the serum was aliquoted and stored at -80 °C. The liver and colon were excised, rinsed with ice-cold saline, blotted dry, snap-frozen in liquid nitrogen, and stored at -80°C. Liver samples designated for histopathology were fixed in 10 % neutral buffered formalin-phosphate solution.

The tissue was washed with pre-cooled PBS to remove residual blood, weighed, and cut into pieces. The cut tissue was combined with the corresponding volume of PBS (1 g: 9 mL) in a glass homogenizer, ground thoroughly on ice, and subsequently centrifuged at 5000 rpm for 10 min. The supernatant was collected for the analysis of blood lipid and inflammatory indicators.

### 2.4 Analysis of biochemical indices

We determined the levels of TC, TG, LDL-C, VLDL-C, HDL-C, AST, and ALT in the serum and liver supernatants in accordance with accepted laboratory procedures.

### 2.5 ELISA detection of inflammatory markers in the supernatant of colon tissue

An enzyme-linked immunosorbent assay (ELISA) was used to measure the amounts of inflammatory markers in the colon tissue supernatant, including IL-6, TNF-α, IFN-γ, IL-4, IL-10, and SIgA, according to the supplied instructions. Optical density at 450 nm (OD450) was measured using a fully automated enzyme-linked immunosorbent assay (ELISA) reader. Concentration values were determined using a pre-defined standard curve.

### 2.6 Liver Oil red O staining

Fresh liver tissues were embedded using optimal cutting temperature (OCT) chemicals and stored at −20°C. The tissue blocks were sliced into slices, each approximately 5 μm thick, using a freezing microtome. Oil Red O was used to stain the sections for 15 min, followed by 30 s of hematoxylin and eosin staining. Following the staining procedure, the slices were washed under running water and adhered to glass slides. The resultant sections (Hwang et al., 2023).

### 2.7 Quantitative polymerase chain reaction

After total RNA extraction using TRIzol reagent, samples from the colon and liver were treated with DNase I according to the manufacturer’s instructions. Next, we determined the RNA concentrations in each sample. The PrimeScript RT Reagent Kit was used to prevent genomic DNA contamination before reverse transcription. In order to synthesize cDNA, Prime Script Enzyme Mix 1, RT Primer Mix, and 5 × Prime Script Buffer were utilized. After 15 min of reverse transcription at 37°C, the samples were incubated at 85°C for 3 s. Sangon Biotech Co., Ltd., used NCBI to design and manufacture gene-specific primers, the sequences of which are listed in Table 1. We performed the experiments according to the manufacturer’s instructions. Gene expression levels in the control and treatment groups were compared using the 2^–ΔΔ*Ct*^ method, where Ct represents Ct (target) - Ct (β-actin). A housekeeping gene was selected to normalize the transcript levels of the target genes (Li L. et al., 2025).

**TABLE 1:** Primer sequences for RT-PCR.

Primer name	Sequence (5’-3’)	Sequence (3’-5’)
β-actin	GTGACGTTGACATCCGTAAAGA	GTAACAGTCCGCCTAGAAGCAC
SREBP2	GATGGATGAGAGCAGCGAGC	CTCTCCCACTTGATTGCTGACA
HMG-COAR	CAAGTACATTCTGGGTATTGCTGG	TAAGCCTGTCAGTTCTTTGTCG
ABCA1	AGTCCATCGTGTCTCGCCTGT	GGGATGCTTGATCTGCCGTA
ABCG5	ACGTTGCGATACACAGCGA	TTGGGCTGCGATGGAAACTC
ABCG8	CAACTGCTGCCCAACCTGAC	ATTACGTCTTCCACCCGTTTGT
LCAT	CTGTCTGCATGTGCTCCACTTCT	TGGTTATGCGCTGCTCCTCTT
LDLR	TGATGGAGACCGAGATTG	GCTGCGATGGATACACT
APOA1	GCCAACAGCTGAACCTGAATCTC	GTTTCACTTCCTCTAGGTCCTTGTTC
INF-γ	CTCAAGTGGCATAGATGTGGAAG	TGACCTCAAACTTGGCAATACTC
IL-6	CCCCAATTTCCAATGCTCTCC	CGCACTAGGTTTGCCGAGTA
TNF-α	CCTCACACTCACAAACCACCAA	CTCCTGGTATGAGATAGCAAATCG
TLR4	TGAGGACTGGGTGAGAAATGAGC	CTGCCATGTTTGAGCAATCTCAT
NF-κB(p65)	CTGACCCCTGTCCTCTCACAT	ATACACCTCAATGTCTTCTTTCTGC
SIgA	CGGAAGGGAAGTAATCGTGAAT	AGAAATCCCACCATCTACCCAC
IL-4	GATAAGCTGCACCATGAATGAGT	CCATTTGCATGATGCTCTTTAGG
IL-10	AATAAGCTCCAAGACCAAGGTGT	CATCATGTATGCTTCTATGCAGTTG

### 2.8 16S rRNA sequencing

The procedures are outlined in a previous study (Almousa et al., 2025). Total microbial community DNA was extracted from samples of diverse origins using a modified cetyltrimethylammonium bromide (CTAB) protocol. DNA integrity was assessed by 1% agarose gel electrophoresis (120 V, 30 min), and purity was confirmed using a NanoDrop™ spectrophotometer (A260/280 = 1.8–2.0; A260/230 > 1.5). The V3-V4 hypervariable region of the 16S rRNA gene was amplified with universal primers 341F (5’-CCTACGGGNGGCWGCAG-3’) and 806R (5’-GACTACHVGGGTATCTAATCC-3’) in a 25 μL reaction: 98°C for 30 s initial denaturation; 35 cycles of 98°C for 10 s, 54°C for 30 s, 72°C for 45 s; and final extension at 72°C for 10 min. Amplicons were verified on a 2 % agarose gel, purified with AMPure XP beads (Beckman Coulter), and analyzed on an Agilent 2100 Bioanalyzer (main peak 450–470 bp). Libraries were quantified by qPCR using the KAPA Library Quantification Kit (Illumina) and accepted when the concentration was ≥ 2 nM. The indexed libraries were pooled in equimolar ratios, denatured with NaOH, and sequenced on an Illumina NovaSeq 6000 platform (2 × 250 bp paired-end reads). Raw reads were processed as follows: paired-end reads were merged with FLASH v1.2.8 (≥ 10 bp overlap, < 10 % mismatches); low-quality sequences (Q < 20, length < 50 bp) were trimmed with Fqtrim v0.94; and chimeras were removed using Vsearch v2.3.4 (UCHIME_ref algorithm). Denoising, error correction, and amplicon sequence variant (ASV) inference were performed using QIIME2 DADA2 (denoise-paired), discarding singleton ASVs (prevalence < 2).

Alpha-diversity indices (Shannon, Simpson, Chao1, and ACE) and beta-diversity (Bray–Curtis distances) were calculated from the ASV table. Ordinations (PCA and PCoA) and statistical tests (PERMANOVA/ANOSIM) were used to assess community dissimilarity. Taxonomic assignment was performed using the SILVA Release 138 NT-16S database at a confidence threshold of 0.7. The relative abundances at the phylum and genus levels were summarized for each sample. Differential abundance among biologically replicated groups was tested using the Kruskal–Wallis test (*p* < 0.05). Indicator taxa were identified and visualized as bubble plots using R v3.4.4. Spearman’s correlation coefficients were computed to examine the associations between blood parameters and microbial abundance, and co-occurrence networks at the phylum and genus levels were constructed using R packages.

### 2.9 Statistical assessment

Data analysis was conducted using GraphPad Prism10.0, and the results are expressed as mean ± SEM. One-way ANOVA was employed for comparisons among three or more groups, followed by Tukey’s multiple comparison test to ascertain statistical significance. A *p*-value < 0.05 was considered significant (*p < 0.05, **p < 0.01, ***p < 0.001).

## 3 Results

### 3.1 LBT’s impact on mice’s serum biochemical indices

**FIGURE 2:**
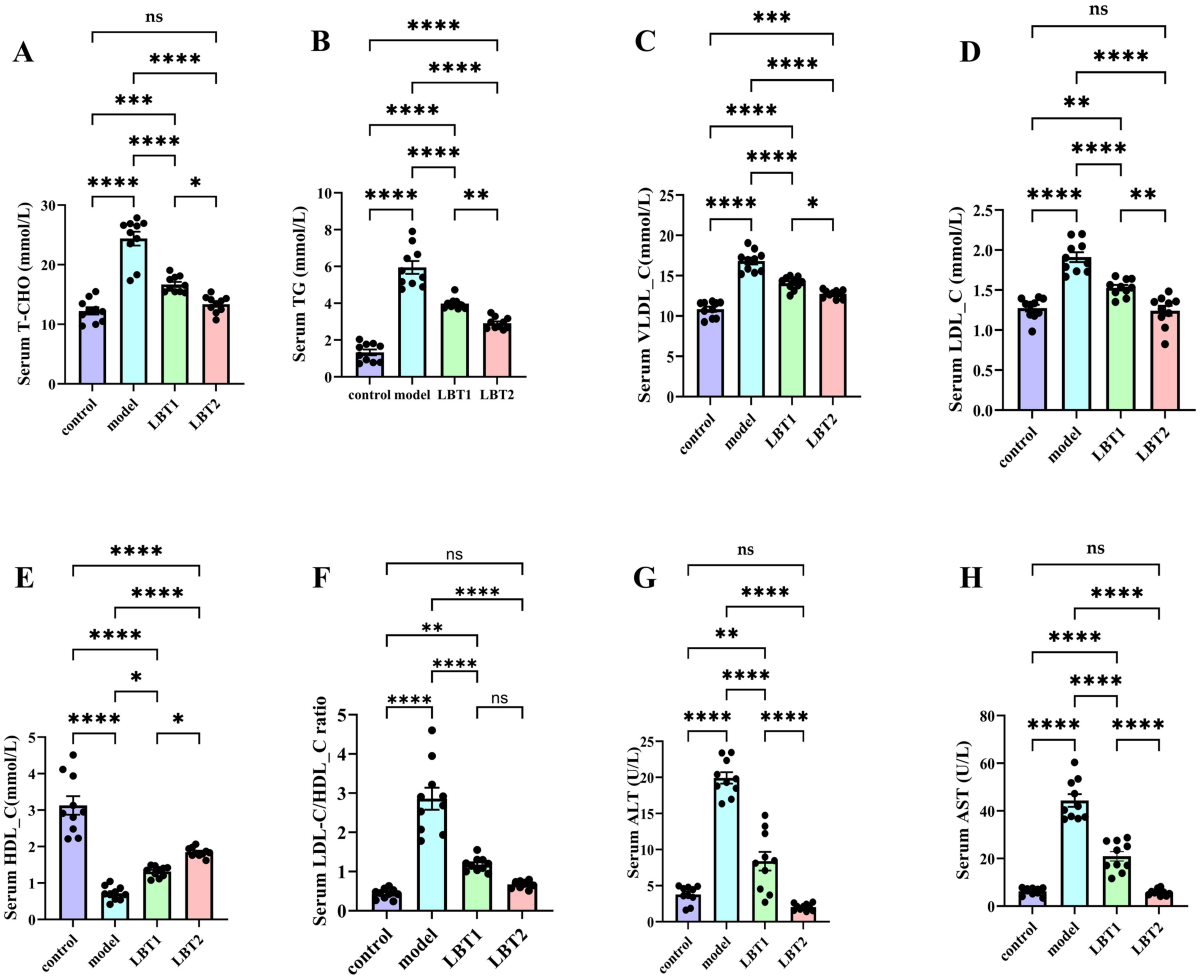
Effect of LBT on serum lipid metabolism indicators in hyperlipidemic mice. **(A)** Serum total cholesterol (T-CHO) **(B)** Serum TG levels. **(C)** Serum VLDL-C levels were measured. **(D)** Serum LDL-C level. **(E)** Serum HDL-C levels were measured. **(F)** Serum LDL-C/HDL-C ratio. **(G)** Serum ALT level. **(H)** Serum AST level. *n=10*. *P*-values were determined using a two-way ANOVA. ^ns^*p* > 0.05, *p < 0.05, **p < 0.01, ***p < 0.001, and ****p < 0.0001.

Figure 2 shows that LBT decreased the serum metabolic indicators in hyperlipidemic mice. Serum HDL-C levels decreased in the model group, while TC, TG, LDL-C, VLDL-C, and the LDL-C/HDL-C ratio were significantly higher than in the control group. Compared to the model group, the results exhibited a strong dose-response relationship, with LBT significantly lowering TC, TG, LDL-C, VLDL-C, and the LDL-C/HDL-C ratio, while increasing the HDL-C content (Figures 2A–E). Mice treated with Triton WR-1339 had significantly higher serum AST and ALT levels than those in the control group. Following an increase in LBT concentrations, mice in the LBT group had noticeably lower serum AST and ALT levels than those in the model group. These results show that LBT significantly reduced Triton WR-1339-induced hyperlipidemia.

### 3.2 LBT’s effect on mice’s hepatic parameters

**FIGURE 3:**
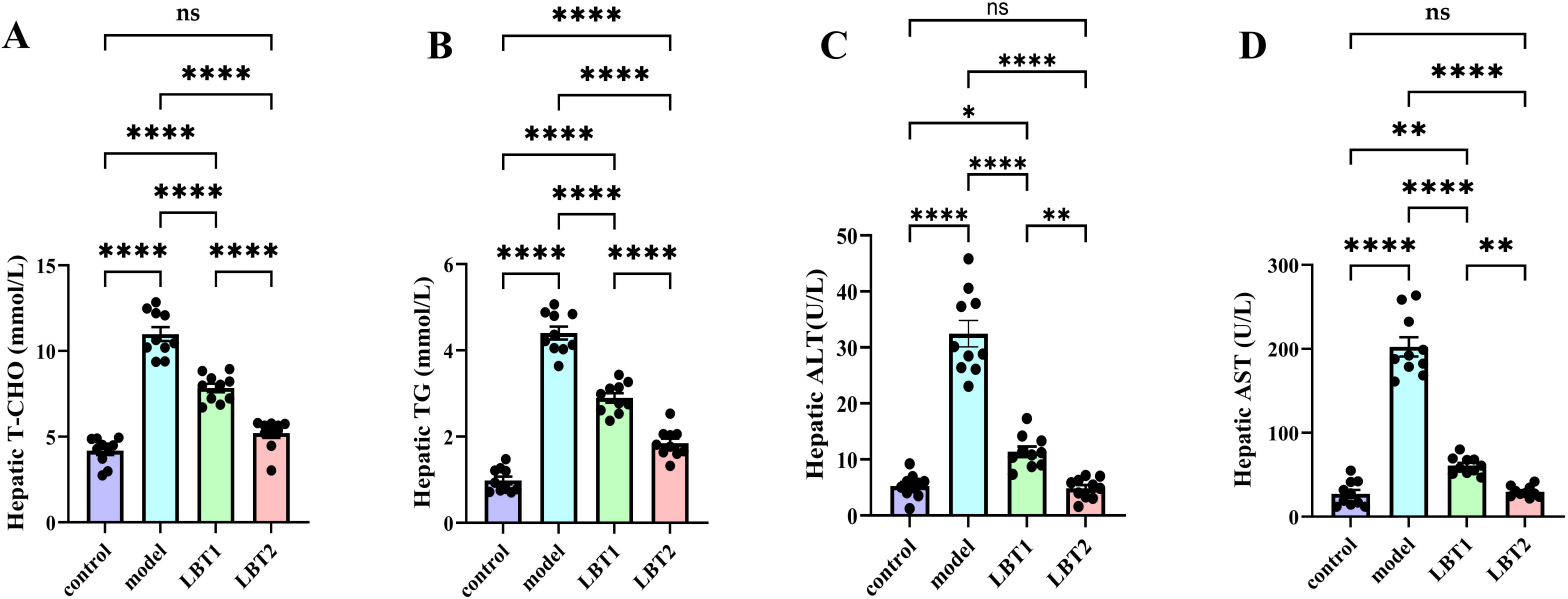
Effect of LBT on hepatic lipid metabolism indicators in hyperlipidemic mice. **(A)** Level of hepatic T-CHO. **(B)** Hepatic TG levels. **(C)** Hepatic ALT levels. **(D)** Level of hepatic AST. *n=10*. *P*-values were determined using a two-way ANOVA. ^ns^*p* > 0.05, *p < 0.05, **p < 0.01, and ****p < 0.0001.

Triton WR-1339 significantly increased TG and TC levels in the livers of mice compared to the control group (Figures 3A, B). Pretreatment with varying concentrations of LBT for 10 days resulted in considerably lower levels of triglycerides and total cholesterol in the liver tissue of mice than in the Triton WR-1339 group, suggesting a dose-dependent effect. Mice administered Triton WR-1339 had elevated AST and ALT levels in the liver compared to those in the control group (Figures 3C, D). A marked reduction in AST and ALT levels was observed in the livers of hyperlipidemic mice administered various dosages of LBT. According to the results, LBT was able to ameliorate the problems with liver lipid metabolism and the damage caused by Triton WR-1339.

### 3.3 Impact of LBT on hepatic pathology sections

**FIGURE 4:**
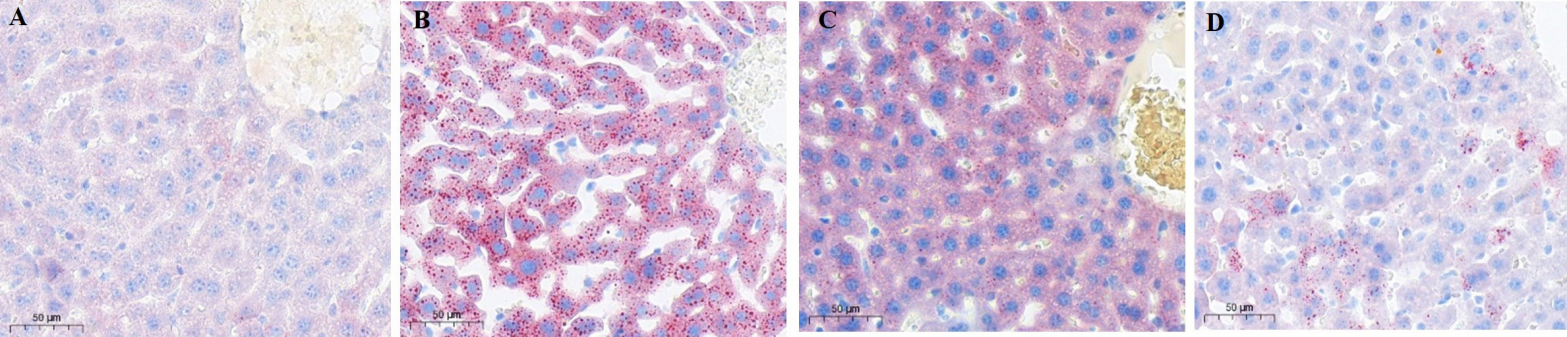
Oil red staining image of mouse liver slices (×20 magnification). **(A)** Control group. **(B)** Model group. **(C)** LBT1 group. **(D)** LBT2 mice. Scale bar: 50 μm.

Liver pathology concerning LBT was assessed using Oil Red O staining, which imparts a blue hue to the cell nuclei and a red hue to lipid droplets. Hepatocyte nuclei in the control group stained blue were distinctly observable with minimal red lipid droplets. In contrast, the model group showed a significant accumulation of red lipid droplets. The LBT1 group displayed a moderate quantity of red lipid droplets relative to the control and model groups, whereas the LBT2 group demonstrated a reduced number of red lipid droplets (Figures 4A–D).

### 3.4 The effect of LBT on genes linked to lipid metabolism in mouse hepatic tissue

**FIGURE 5:**
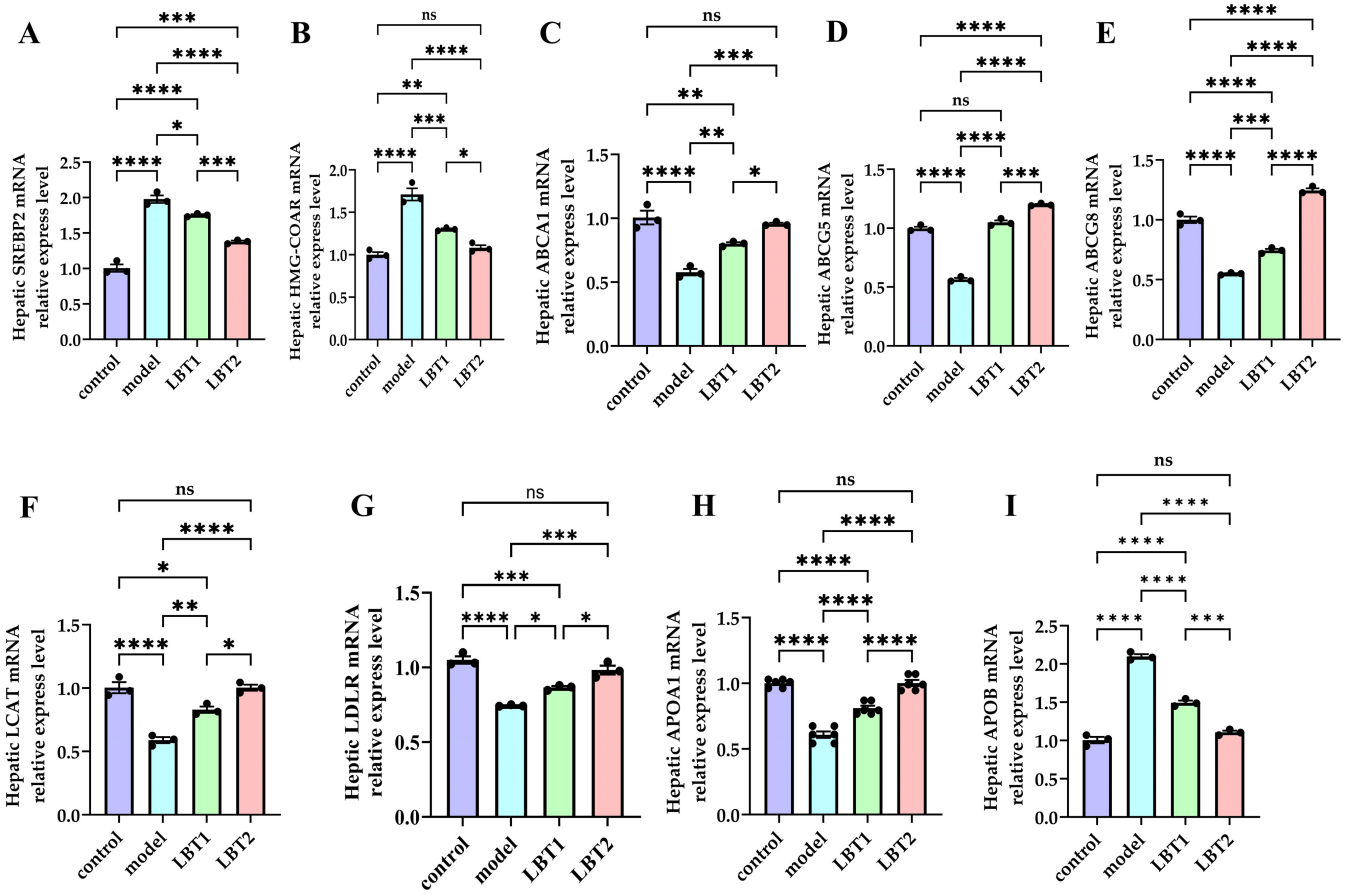
Changes in gene expression related to cholesterol metabolism in the mouse liver. **(A)** Level of hepatic SREBP2 mRNA. **(B)** Level of hepatic HMGCAR mRNA. **(C)** Level of hepatic ABCA1 mRNA. **(D)** Level of hepatic ABCG5 mRNA. **(E)** Level of hepatic ABCG8 mRNA. **(F)** Level of hepatic LCAT mRNA. **(G)** Level of hepatic LDLR mRNA. **(H)** Level of hepatic APOA1 mRNA. **(I)** Level of hepatic APOB mRNA. Data are representative of three independent experiments (*n=3*). *P*-values were determined using a two-way ANOVA. ^ns^*p* > 0.05, *p < 0.05, **p < 0.01, ***p < 0.001, and ****p < 0.0001.

Compared to the control group, the mice in the model group demonstrated markedly higher mRNA levels of SREBP2 and HMGCR in their livers (Figures 5A, B), indicating an enhanced cholesterol synthesis. Simultaneously, the mRNA levels of ABCA1, ABCG5, ABCG8, LCAT, LDLR, and APOA1 were markedly diminished in the hepatic tissues of these mice (Figures 5C–I), indicating suppression of cholesterol efflux and reverse cholesterol transport. Compared with the model group, mice in the LBT1 and LBT2 groups had significantly reduced mRNA levels of SREBP2 and HMG-COAR in their livers (Figures 5A, B), with the LBT2 group displaying a more pronounced effect than the LBT1 group. Furthermore, the mRNA levels of ABCA1, ABCG5, ABCG8, LCAT, LDLR, and APOA1 were markedly elevated (Figures 5C–I), with the LBT2 group demonstrating a more pronounced effect than the LBT1 group. These findings indicate that LBT effectively mitigates hepatic lipid metabolic disorders by downregulating the expression of genes related to cholesterol synthesis (SREBP2 and HMGCR) and upregulating the expression of genes involved in cholesterol efflux and reverse cholesterol transport (ABCA1, ABCG5, ABCG8, LCAT, LDLR, and APOA1). The enhanced efficacy of LBT2 compared to that of LBT1 suggests that the effects of LBT are contingent on dosage.

### 3.5 LBT’s effect on inflammatory factors associated with the colon in MAFLD mice

**FIGURE 6:**
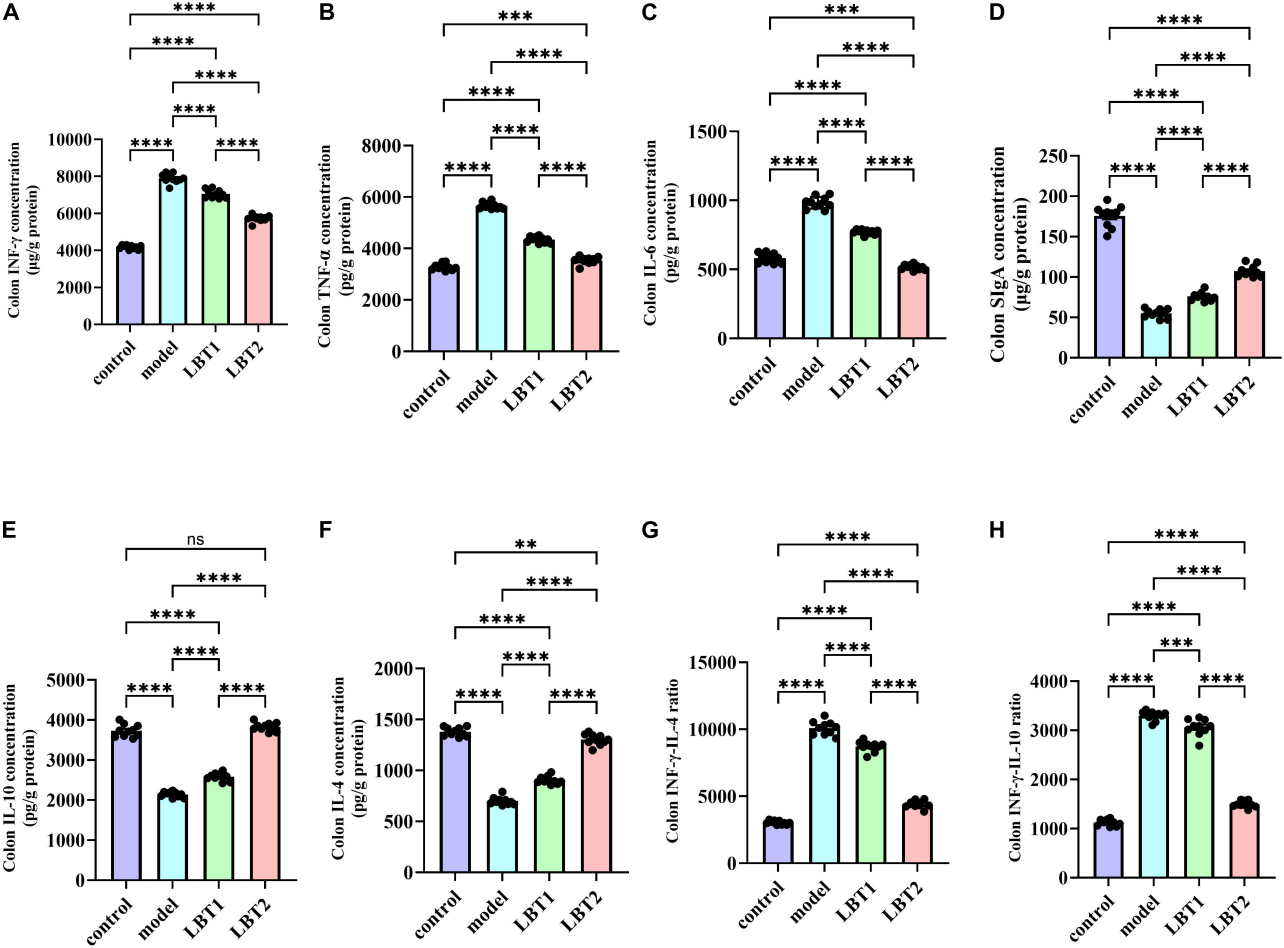
Changes in inflammation-related gene expression in the mouse colon. **(A)** Colonic INFγ content. **(B)** Colonic TNF-α levels. **(C)** Colonic IL-6 levels. **(D)** Colonic SIgA contents. **(E)** Colonic IL-10 contents. **(F)** Colonic IL-4 contents. **(G)** Colonic INFγ/IL-4 ratio. **(H)** Colonic INFγ/IL-4 ratio. *n=10*. *P*-values were determined using a two-way ANOVA.^ns^*p* > 0.05, **p < 0.01, ***p < 0.001, and ****p < 0.0001.

Figure 6 shows the results of our ELISA-based determination of the levels of components associated with colonic inflammation in the mice. The levels of IFN-γ, TNF-α, and IL-6 in the colon were significantly higher in the Triton WR-1339 group than in the control group (Figures 6A–C). Figures 6D, E shows that the levels of IL-4 and IL-10, which are anti-inflammatory cytokines, were considerably lower. Figures 6F, G show a considerable increase in the INF-γ/IL-4 and INF-γ/IL-10 ratios, whereas Figure 6H shows a noticeably decreased SIgA content compared with that in the control group. The levels of IFN-γ, TNF-α, and IL-6 were significantly reduced by LBT compared to those in the model group. This inhibitory effect became more apparent as the LBT concentration increased (Figures 6A–C). In addition, LBT use led to an increase in IL-4 and IL-10 levels and a decrease in the INF-γ/IL-4 and INF-γ/IL-10 ratios (Figures 6D–G), and a notable increase in SIgA content (Figure 6H), with the effect becoming more pronounced as the LBT concentration increased. These results indicate that Triton WR-1339 altered the Th1/Th2 cell balance in favor of Th1, decreased colonic mucosal immune function, and dramatically increased the levels of intestinal inflammation in MAFLD mice. In hyperlipidemic mice, LBT restored the balance of Th1/Th2 cells and improved the colon’s anti-inflammatory status and mucosal immune function by lowering the levels of inflammatory cytokines, including IFN-γ, TNF-α, and IL-6, increasing the levels of anti-inflammatory cytokines, such as IL-4 and IL-10, and increasing SIgA content.

### 3.6 LBT’s impact on inflammatory factor gene expression in MAFLD mice’s colon

**FIGURE 7:**
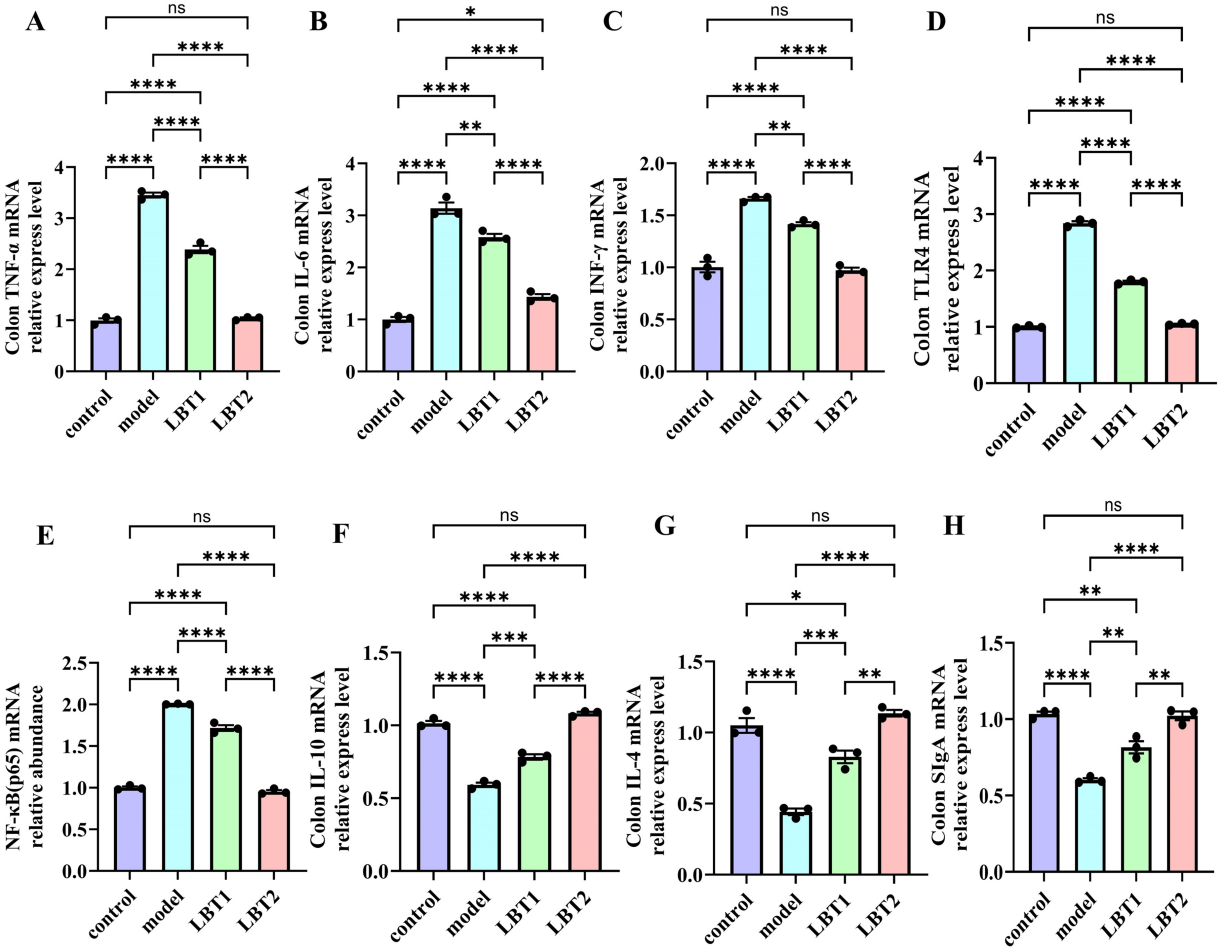
Changes in inflammation-related gene expression in the mouse colon. **(A)** Level of colonic TNF-γ mRNA. **(B)** Level of colonic IL-6 mRNA. **(C)** Colonic INF-γ mRNA levels. **(D)** Level of colonic TLR4 mRNA. **(E)** Level of colonic NF-γB(p65) mRNA. **(F)** Level of colonic IL-10 mRNA. **(G)** Content of colonic IL-4 mRNA. **(H)** Content of colonic SIgA mRNA. *n=3*. *P*-values were determined using two-way ANOVA, ^ns^*p* > 0.05, *p < 0.05, **p < 0.01, ***p < 0.001, and ****p < 0.0001.

The results regarding inflammation-related gene components in the mouse colon are shown in Figure 7. The model animals exhibited substantially higher expression levels of INF-γ, TNF-α, IL-6, TLR4, and NF-κB(p65) than those of the control group (Figures 7A–E). Conversely, the concentrations of IL-4, IL-10, and IgA were significantly decreased (Figures 7F–H). Compared to the model group, the gene expression of INF-γ, TNF-α, IL-6, TLR4, and NF-κB(p65) was significantly reduced with LBT treatment. This inhibitory effect was more pronounced at higher LBT concentrations (Figures 7A–E). Furthermore, LBT administration considerably increased the expression of IL-4, IL-10, and SIgA, with the effect of being more pronounced at higher LBT concentrations (Figures 7F–H). These results suggest that LBT may reduce inflammation in hyperlipidemic mice by inhibiting the TLR4/NF-κB (p65) signaling pathway and enhancing immunological responses in the colonic mucosa of hyperlipidemic mice.

### 3.7 LBT’s effect on mice’s gut microbiota diversity

**FIGURE 8:**
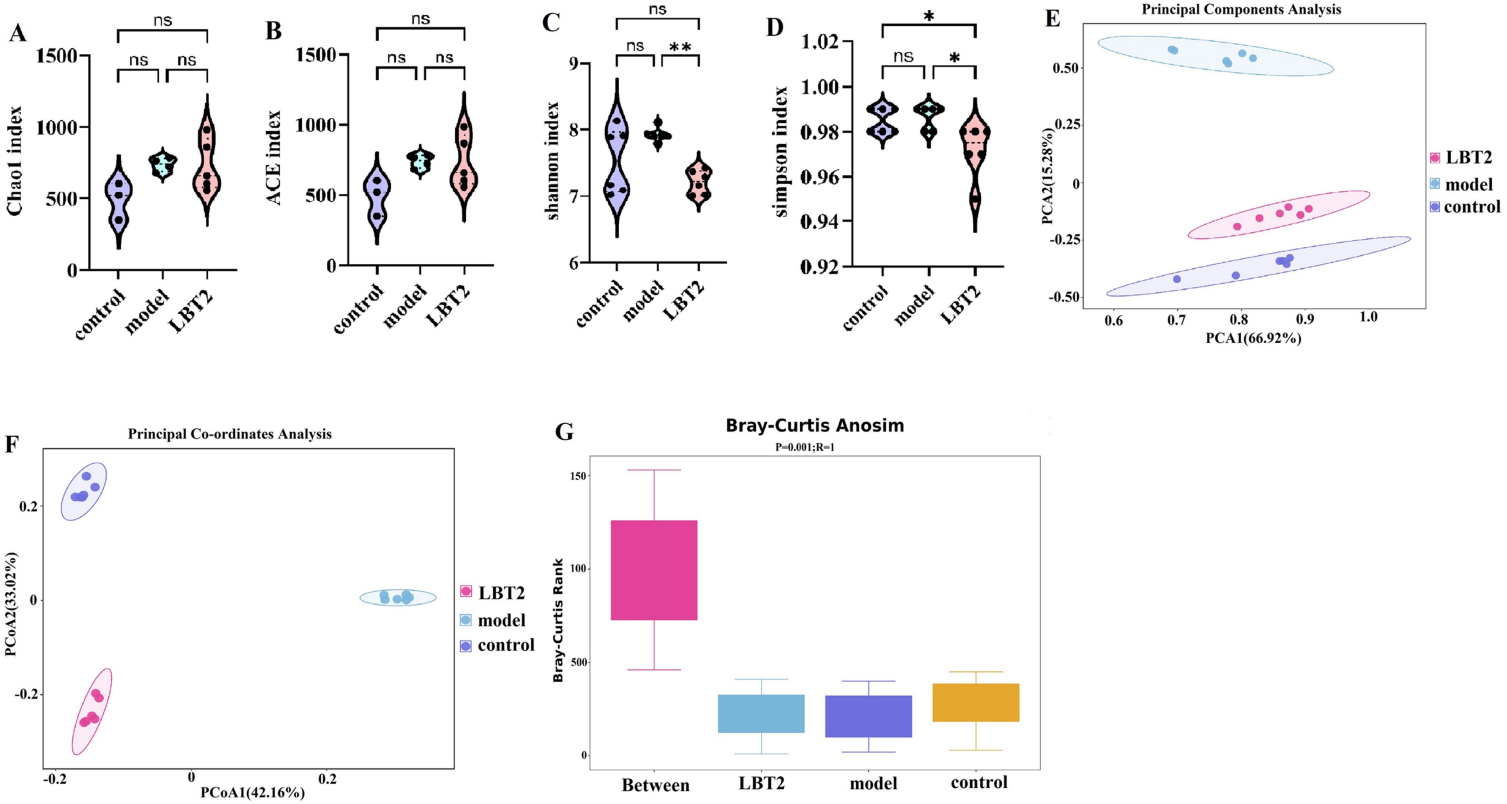
Changes in the gut microbiota diversity of mice. **(A)** Chao1 index. **(B)** ACE index. **(C)** Shannon index. **(D)** Simpson index. **(E)** Principal coordinate analysis (PCA) of 16S sequences from 18 samples using the Bray-Curtis distance of fecal microbiota among the three groups, which showed distinct separation of samples. **(F)** Principal Coordinate Analysis (PCoA) of 16S sequences from 18 samples using the Bray-Curtis distance of fecal microbiota among the three groups, which showed distinct separation of samples. **(G)** Anosim analysis using Bray-Curtis-anosim. *n=6*. Data are mean ± SEM. *P*-values were determined using two-way ANOVA, ^ns^*p* > 0.05, *p < 0.05, and **p < 0.01.

The fecal microbiota types of the model and LBT2 groups of mice were not substantially different, as depicted in Figures 8A, B. Additionally, Figures 8C, D suggest that the Uniformity of Gut Microbiota in the LBT2 group was substantially lower than that in the model group. As shown in Figure 8E, we conducted unsupervised PCA on all the samples. The microbial communities of the control, model, and LBT2 groups were successfully divided into various regions by the first two explanatory factors. The observation that the microbial populations of the control, model, and LBT2 groups were significantly different was further supported by further PCoA, which is shown in Figure 8F. This highlights the significant differences in species composition between these groups. Intergroup differences were more noticeable than intragroup differences, according to analysis of similarities (ANOSI) (Figure 8G). Additionally, Permanova (Table 2) confirmed notable differences in sample interpretation between the groups, which was consistent with the ANOSIM results. Significant differences between and within the three mouse groups’ intestinal microbiota were revealed by the observations, suggesting that the degree of species diversity similarity can be clearly explained using QIIME software for beta diversity analysis.

**TABLE 2:** Adonis analysis.

	df	sum of Sqs	R^2^	F	Pr ( > F)
Group	2	1.49	0.75	33.34	0.00
Residual	15	0.50	0.25		
Total	17	1.99	1.00		

### 3.8 LBT’s effect on the distribution of phyla and genera of the MAFLD mice’s gut microbiota

**FIGURE 9:**
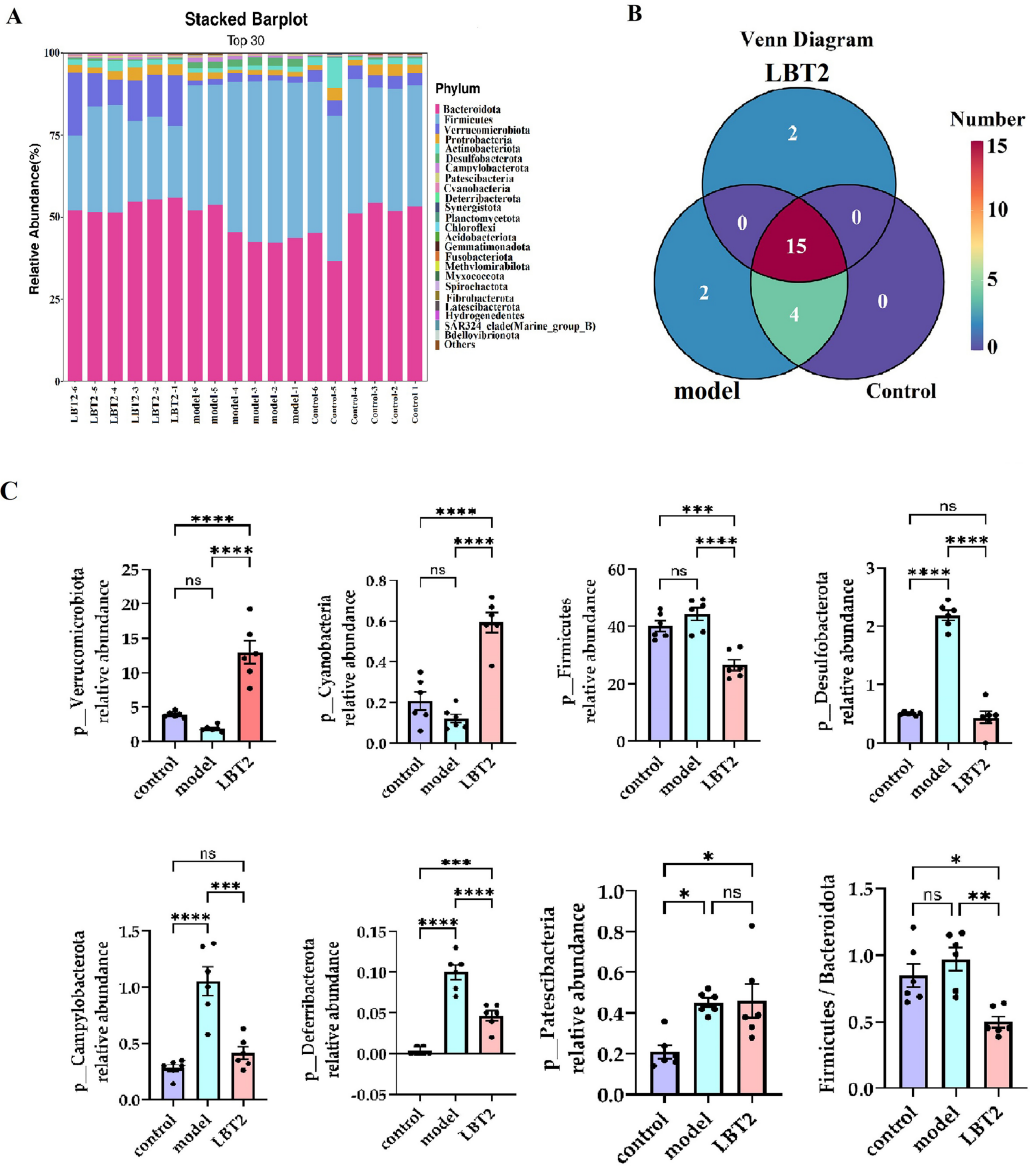
Changes in the distribution of gut microbiota phyla in mice. **(A)** Horizontal bar chart of the mouse gut microbiota phylum. **(B)** Venn diagram of the mouse gut microbiota at the phylum level. **(C)** Differential analysis of the gut microbiota among different phyla. *n=6*. Data are mean ± SEM. *P*-values were determined using two-way ANOVA, ^ns^*p* > 0.05, *p < 0.05, **p < 0.01, ***p < 0.001, and ****p < 0.0001.

The top 30 phyla distributions for the control, model, and LBT2 groups are shown in Figure 9A: *Firmicutes* accounted for 37.02%, *Verrucomicrobiota* accounted for 6.22 %, and *Bacteroidetes* accounted for 49.73%. The control, model, and LBT2 groups shared 15 phyla, whereas the control and model groups shared four phyla. The model and LBT2 groups had two distinct phyla (Figure 9B). *Deferribacterota*, *Desulfobacterota*, *Campylobacterota*, and *Patescibacteria* were substantially more abundant in the model group than in the control group (Figure 9). *Verrucomicrobiota* and *Cyanobacteria* levels were (Figure 9) significantly higher in the LBT2 group than in the model group, whereas *Deferribacterota*, *Desulfobacterota*, *Campylobacterota*, and the *Firmicutes*/*Bacteroidota* ratio were significantly lower (Figure 9C).

**FIGURE 10:**
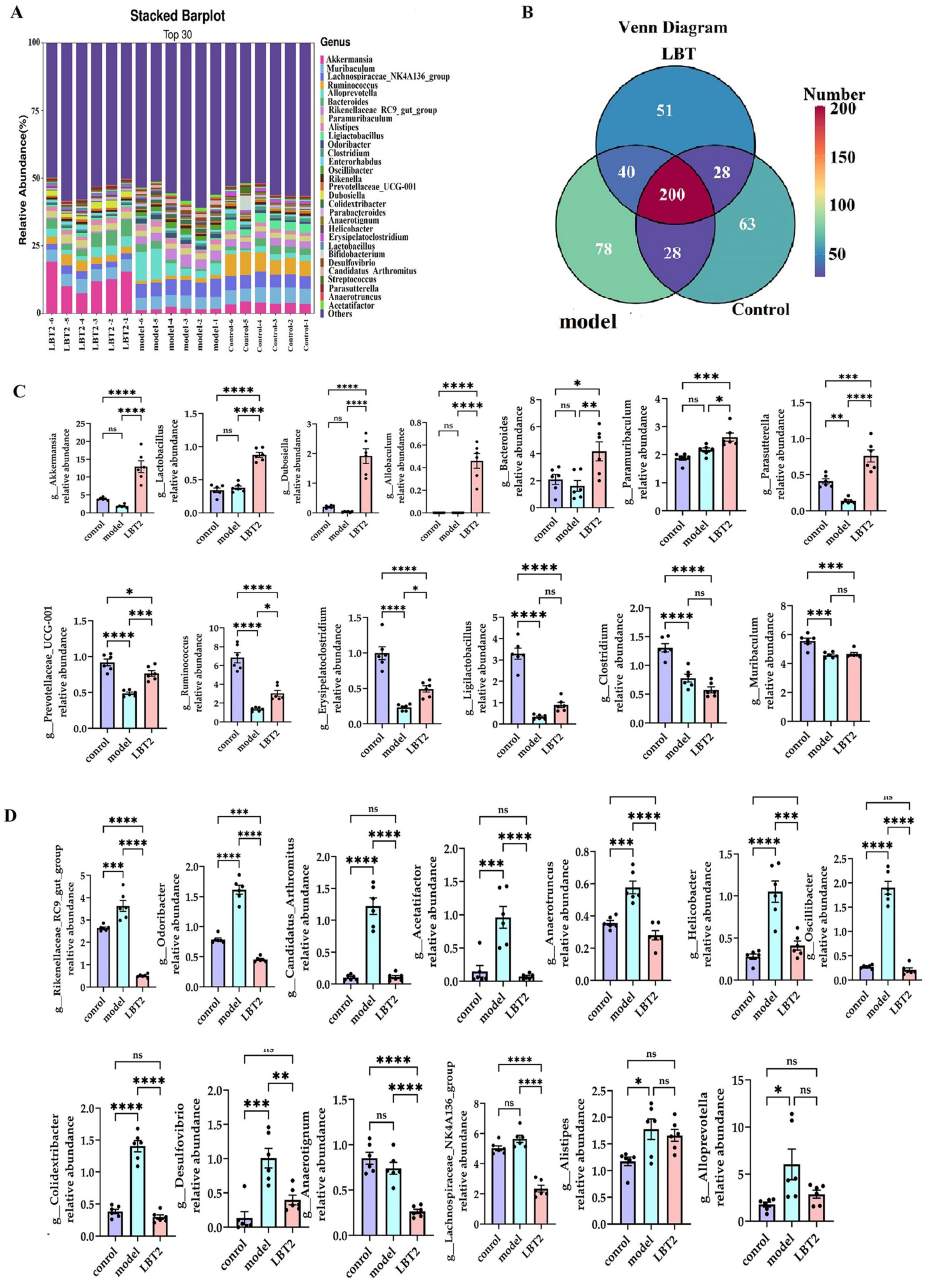
Changes in the distribution of gut microbiota genera in mice. **(A)** The Horizontal bar chart of mouse gut microbiota genus. **(B)** Venn diagram of mouse gut microbiota at the genus level. **(C)** Differential analysis of gut microbiota among genera, *Akkermansia*, *Lactobacillus*, *Dubosiella*, *Allobaculum*, *Bacteroides*, *Paramuribaculum*, *Parasutterella*, *Prevotellaceae_UCG-001*, *Ruminococcus*, *Erysipelatoclostridium*, *Ligilactobacillus*, *Clostridium*, and *Muribaculum*. **(D)** Differential analysis of gut microbiota among genera *Rikenellaceae_RC9_gut_group*, *Odoribacter*, *Candidatus_Arthromitus*, *Acetatifactor*, *Anaerotruncus*, *Helicobacter*, *Oscillibacter*, *Colidextribacter*, *Desulfovibrio*, *Anaerotignum*, *Alistipes*, *Lachnospiraceae_NK4A136_group*, and *Alloprevotella*. *n* = 6. Data are mean ± SEM. *P*-values were determined using two-way ANOVA, ^ns^*p* > 0.05, *p < 0.05, **p < 0.01, ***p < 0.001, and ****p < 0.0001.

As shown in Figure 10A, the control, model, and LBT2 groups shared 200 common genera. There were 40 genera shared between the model and LBT2 groups, 28 genera shared by the control group, and 28 genera shared between the model and LBT2 groups. The control group had 63 genera, the model group had 78 genera, and the LBT2 group had 51 genera. Figure 10B shows the top 30 genera in the three groups. The model group had significantly lower levels of *Ruminococcus*, *Ligilactobacillus*, *Erysipelatoclostridium*, *Parasutterella*, *Clostridium*, and *Prevotellaceae_UCG-001* than those in the control group (Figure 10C). Conversely, the populations of *Odoribacter*, *Rikenellaceae_RC9_gut_group*, Desulfovibrio, *Oscillibacter*, *Colidextribacter*, *Anaerotruncus*, *Helicobacter*, *Candidatus_Arthromitus*, *Acetatifactor*, and *Alloprevotella* are markedly elevated (Figure 10D). Additionally, while comparing the LBT2 group with the model group, a notable increase in the abundance of *Akkermansia*, *Ruminococcus*, *Dubosiella*, *Erysipelatoclostridium*, *Parasutterella*, *Prevotellaceae_UCG-001*, *Lactobacillus*, *Paramuribaculum*, and *Bacteroides* was observed (Figure 10C). *Odoribacter*, *Lachnospiraceae_NK4A136_group*, *Desulfovibrio*, *Oscillibacter*, *Colidextribacter*, *Anaerotruncus*, *Helicobacter*, *Anaerotignum*, *Candidatus_Arthromitus*, *Acetatifactor*, and *Rikenellaceae_RC9_gut_group* have all significantly dropped (Figure 10D).

These results indicate that hyperlipidemic mice undergo gut microbiota remodeling in response to LBT, with the upregulation of genera such as *Akkermansia*, *Ruminococcus*, *Dubosiella*, *Erysipelatoclostridium*, and *Parasutterella*, and the downregulation of genera such as *Rikenellaceae_RC9_gut_group*, *Odoribacter*, *Desulfovibrio*, *Oscillibacter*, and *Colidextribacter*.

### 3.9 The indicative phyla and genera within each group

**FIGURE 11:**
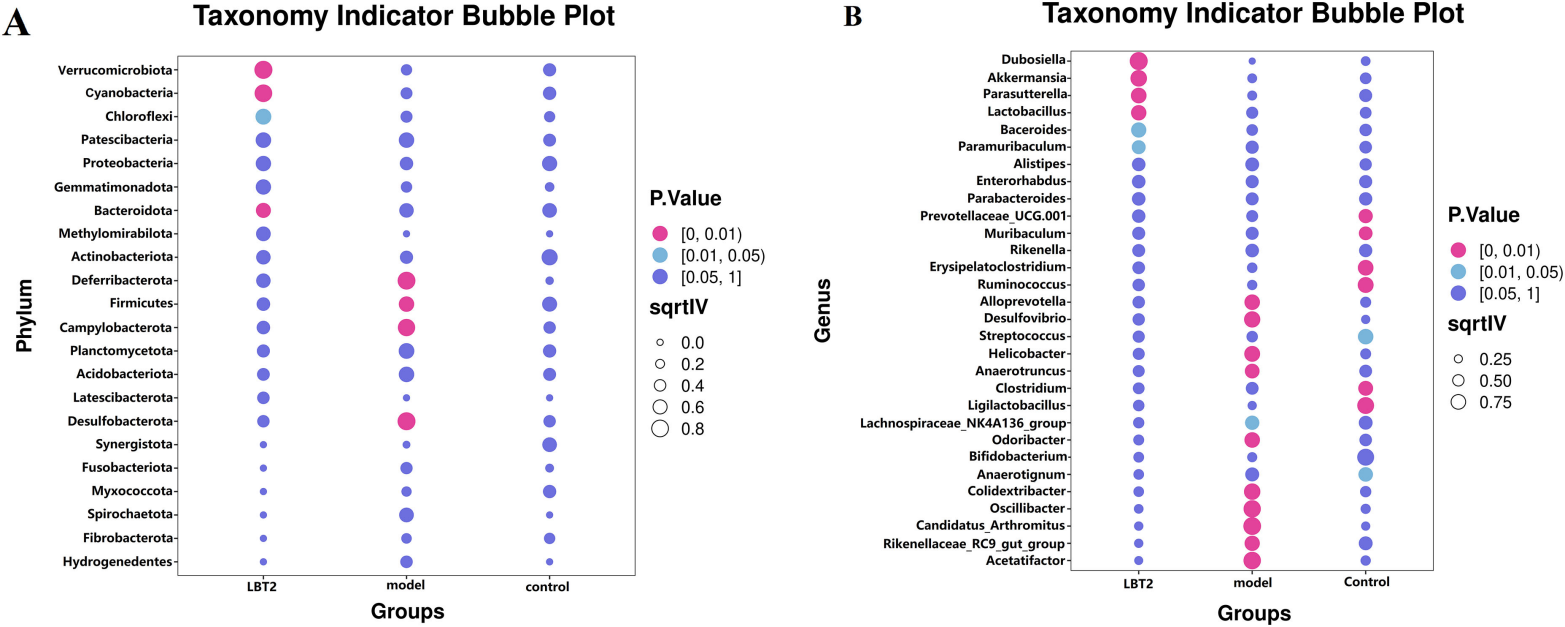
Indicator phyla and genera of gut microbiota in different groups of mice. **(A)** Indicator phyla among the three groups. **(B)** Indicator genera in the three groups. *n=6*. Data are mean ± SEM. *P*-values were determined by two-way ANOVA, *P*[0, 0.01], *P*[0.01, 0.05], and *P*[0.05, 1].

Figure 11A shows that the predominant bacterial phyla in the gut of mice in the model group were *Desulfobacterota*, *Firmicutes*, and *Campylobacterota*, respectively. Conversely, the LBT2 group exhibited *Verrucomicrobiota*, *Cyanobacteria*, and *Bacteroidetes*. Figure 11B shows that the predominant bacterial taxa in the control group were *Clostridium*, *Anaerotruncus*, *Erysipelatoclostridium*, *Rikenella*, and *PrevotellaceaeUCG-001*. The model group was characterized by the presence of *Acetatifactor*, *Rikenellaceae_RC9_gut_group*, *Candidatus_Arthromitus*, *Oscillibacter*, *Odoribacter*, *Desulfovibrio*, *Anaerotruncus*, *Helicobacter*, *Alloprevotella*, Alistipes, and *Lachnospiraceae_NK4A136_ group, whereas* the LBT2 group included *Dub.*

### 3.10 Correlation analysis between phyla and genera of bacteria and indicators of blood lipids and inflammation

**FIGURE 12:**
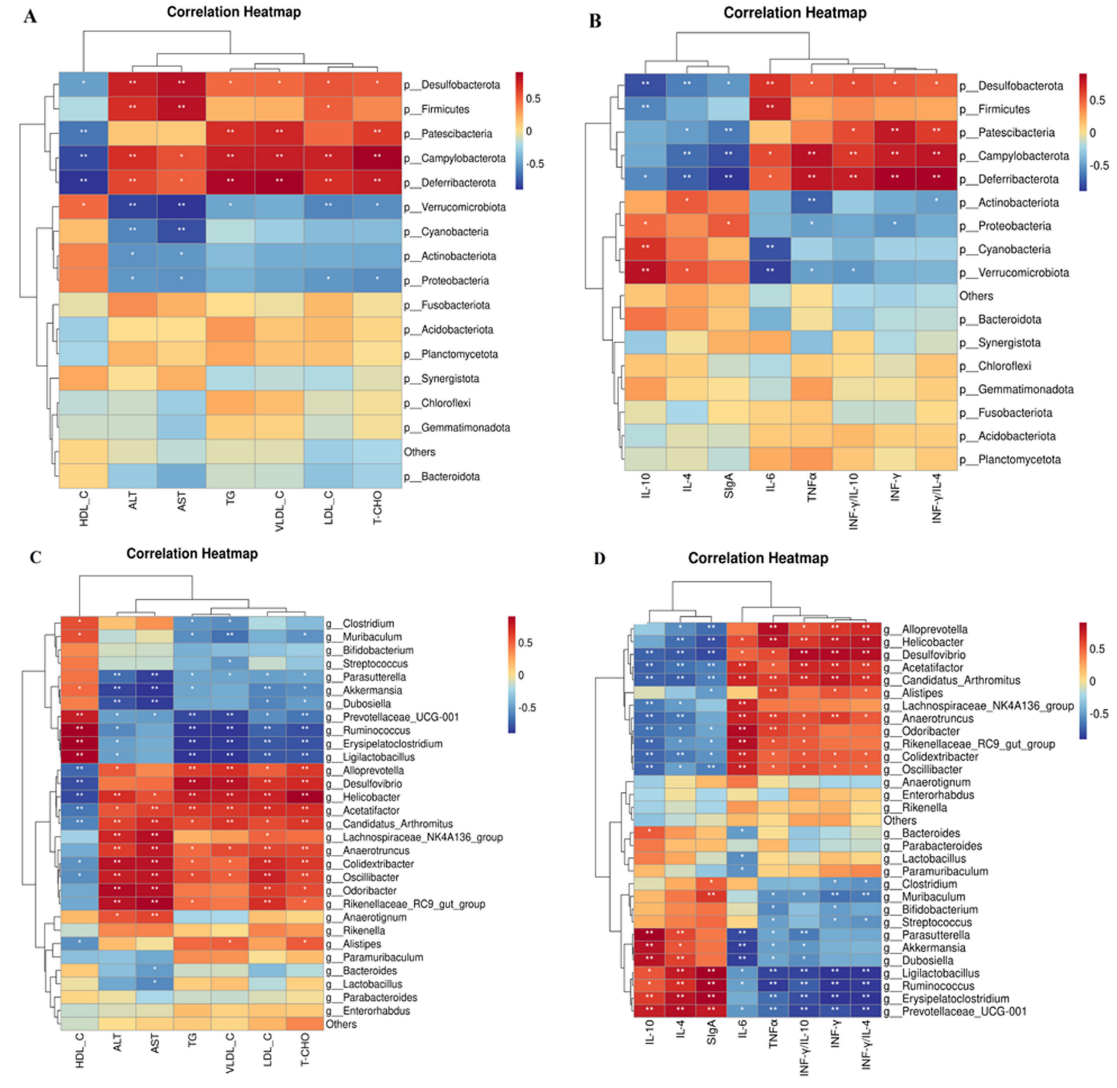
Correlation analysis between phyla, genera, and indicators of blood lipids and inflammation using Spearman’s method. **(A)** Correlation analysis between phyla and blood lipid indicators. **(B)** Correlation analysis between phyla and indicators of colon inflammation. **(C)** Correlation analysis between genera and blood lipid indicators. **(D)** Correlation analysis between genera and indicators of colon inflammation. *n =6*. Data are mean ± SEM. *P*-values were determined using two-way ANOVA, *p < 0.05, **p < 0.01.

Figures 12A, B show the connection between the gut microbiota at the phylum level and indicators of blood lipids and inflammation. The data indicates a good relationship between *Verrucomicrobiota* and HDL-C, as well as a beneficial correlation between IL-10 and SigA. In contrast, there was a negative correlation between the two. Furthermore, *Actinobacteria* had a favorable link with IL-10 but a negative relationship with IL-6 and INF-γ/IL-4 levels. *Cyanobacteria* showed a positive correlation with IL-10 and SIgA levels. *Desulfobacterota* correlated positively with TNF-α, LDL-C, VLDL-C, TC, TG, INF-γ/IL-4,and INF-γ/IL-10, but negatively with HDL-C, IL-10, and SIgA. HDL-C and IL-4 levels were negatively correlated with the abundance of *Patescibacteria*, whereas TC, TG, LDL-C, VLDL-C, IL-6, INF-γ, TNF-α, INF-γ/IL-4, and INF-γ/IL-10 levels were positively correlated. *Campylobacterota* were negatively correlated with high-density lipoprotein cholesterol (HDL-C), IL-4, IL-10, and secretory immunoglobulin A, but positively correlated with INF-γ, TNF-α, IL-6, TC, TG, INF-γ/IL-4,and INF-γ/IL-10. *Deferribacterota* was negatively correlated with HDL-C, IL-4, IL-10, and SIgA but positively correlated with TC, TG, LDL-C, VLDL-C, IL-6, INF-γ, TNF-α, INF-γ/IL-4, and INF-γ/IL-10. *Proteobacteria*, on the other hand, were found to be negatively associated with TC, TG, LDL-C, AST, and ALT levels. Furthermore, *Firmicutes’* abundance was positively correlated with IL-6, LDL-C, AST, and ALT levels.

The relationship between the concentrations of blood lipids and inflammation markers and the genus of intestinal microbiota is shown in Figures 12C, D, respectively. The findings indicated that *Oscillibacter* and *Colidextribacter* had negative associations with HDL-C, IL-10, and SIgA but positive correlations with TC, TG, LDL-C, VLDL-C, ALT, AST, INF-γ, TNF-α, INF-γ/IL-4, and INF-γ/IL-10. TC, TG, LDL-C, VLDL-C, ALT, AST, INF-γ, TNF-α, INF-γ/IL-4, and INF-γ/IL-10 levels were positively associated with *Anaerotruncus abundance*, whereas IL-10 and SIgA levels were negatively correlated. A negative correlation was observed between *Acetatifactor* and *Desulfovibrio* and HDL-C, IL-4, IL-10, and secretory immunoglobulin A. Additionally, *Acetatifactor* exhibited a positive correlation with TC, TG, LDL-C, VLDL-C, ALT, AST, IL-6, INF-γ, TNF-α, INF-γ/IL-4, and INF-γ/IL-10, whereas *Desulfovibrio* exhibited favorable relationships with TC, TG, LDL-C, VLDL-C, ALT, AST, IL-6, INF-γ, TNF-α, INF-γ/IL-4, and INF-γ/IL-10. *Helicobacter* and *Candidatus_Arthromitus* exhibited positive correlations with TC, TG, LDL-C, VLDL-C, ALT, AST, IL-6, INF-γ, TNF-α, INF-γ/IL-4, and INF-γ/IL-10, but negative correlations with HDL-C, IL-4, IL-10, and secretory immunoglobulin A. *Alloprevotella* exhibited positive associations with TC, TG, LDL-C, VLDL-C, ALT, AST, IL-6, INF-γ, TNF-α, INF-γ/IL-4, and INF-γ/IL-10, but negative associations with HDL-C, IL-4, and IL-10. *Alistipes* demonstrated positive correlations with VLDL-C, IL-6, INF-γ, TNF-α, and INF-γ/IL-4 and negative associations with HDL-C and IL-4. HDL-C, IL-10, and SIgA. *Akkermansia* demonstrated negative correlations with TC, TG, LDL-C, ALT, AST, and INF-γ/IL-10, whereas *Prevotellaceae_UCG-001* demonstrated negative associations with TC, TG, LDL-C, VLDL-C, ALT, AST, IL-6, INF-γ, TNF-α, INF-γ/IL-4, and INF-γ/IL-10. *Dubosiella* exhibited a negative correlation with TC, TG, LDL-C, ALT, AST, and INF-γ/IL-10 but a positive correlation with IL-10 and SIgA. *Ligilactobacillus exhibited* positive associations with HDL-C, IL-4, and SIgA and negative associations with TC, TG, LDL-C, ALT, AST, VLDL-C, INF-γ/IL-4, and INF-γ/IL-10. Although *Ruminococcus* and *Erysipelatoclostridium* exhibited negative associations with TC, TG, LDL-C, VLDL-C, AST, ALT, INF-γ/IL-4, and INF-γ/IL-10, they exhibited positive correlations with IL-4, IL-10, and SIgA. HDL-C and SIgA levels were positively correlated with *Clostridium* and *Muribaculum* abundance, whereas TC, TG, LDL-C, VLDL-C, ALT, AST, IL-6, INF-γ, TNF-α, INF-γ/IL-4, and INF-γ/IL-10 levels were negatively correlated with their abundance. Additionally, a unique cluster of bacteria, such as *Odoribacter*, *Lachnospiraceae NK4A136 group*, and *Rikenellaceae_RC9_gutgroup*, exhibited positive correlations with TC, TG, LDL-C, ALT, and AST levels but negative correlations with IL-10 and SIgA levels. *Streptococcus* exhibited a negative correlation with VLDL-C, IL-6, INF-γ, and TNF-α, but a positive correlation with IL-4.

### 3.11 Analysis of the correlation between various microbial communities

**FIGURE 13:**
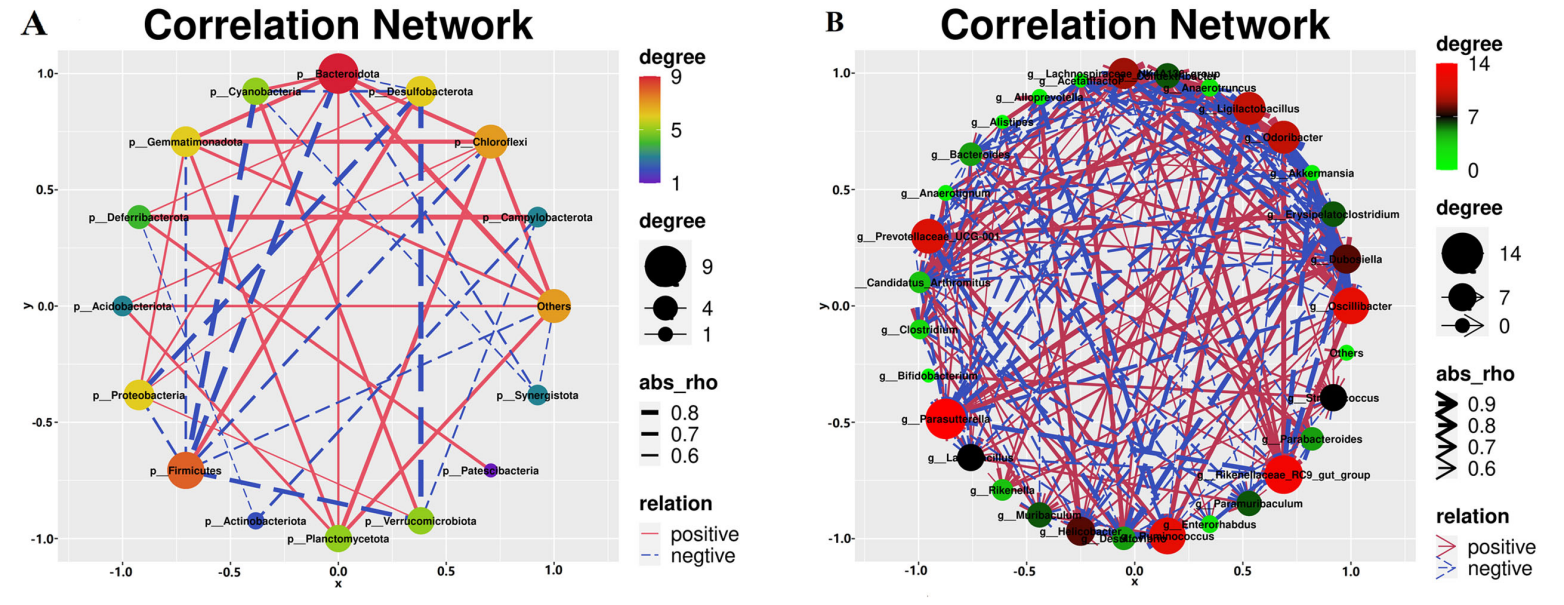
Correlation analysis of the gut microbiota. **(A)** Correlation analysis of different bacterial phyla. **(B)** Correlation analysis of the different bacterial genera. *n=6*.

We conducted a correlation analysis with respect to the bacterial genera and phyla (Figure 13). *Verrucomicrobiota* exhibited a positive correlation with *Cyanobacteria* and *Desulfobacteria* (Figure 13A), whereas *Firmicutes* exhibited a negative correlation with *Campylobacter*. *Cyanobacteria* demonstrated a negative correlation with both *Desulfobacter* and *Firmicutes* and a positive association with *Bacteroidetes*. In addition, *Firmicutes* were negatively correlated with *Bacteroidetes* and *Desulfobacterota*. *Anaerotruncus*, *Anaerotignum*, *Colidextribacter*, *Candidatus_Arthromitus*, *Helicobacter*, *Odoribacter*, and *Oscillibacter* were positively correlated with *Akkermansia*, *Dubosiella*, *Parasutterella*, *Lactobacillus*, as illustrated in Figure 13B. Conversely, *Dubosiella* demonstrates a positive correlation with *Bacteroides*, *Lactobacillus*, and *Parasutterella*, whereas *Anaerotignum*, *Anaerotruncus*, *Colidextribacter*, *Odoribacter*, *Candidatus_Arthromitus*, *Acetatifactor*, and *Oscillibacter* exhibit negative correlations with *Dubosiella*. Moreover, *Bacteroides* is positively correlated with both *Parasutterella* and *Lactobacillus*, as well as with *Anaerotruncus*, *Anaerotignum*, *Colidextribacter*, *Candidatus_Arthromitus*, *Helicobacter*, *Odoribacter*, *Oscillibacter*. Finally, *Lactobacillus* exhibited a positive correlation with *Bacteroides*, *Alistipes*, and *Anaerotignum*, whereas *Colidextribacter* had a negative correlation with *Odoribacter*. *Bacteroides* were positively correlated with *Alistipe* and negatively correlated with *Anaerotruncus*, *Anaerotignum*, *Colidextribacter*, *Candidatus_Arthromitus*, *Odoribacter*.

According to the data, the gut microbiota of the control cohort was primarily composed of the bacterial taxa *Clostridium*, *Anaerotruncus*, *Erysipelatoclostridium*, *Rikenella*, and *Prevotellaceae-UCG-001*. The model cohort, on the other hand, is dominated by the bacterial phyla *Desulfobacterota*, *Firmicutes*, and *Campylobacter*, as well as the genera *Oscillibacter*, *Odoribacter*, *Rikenellaceae_RC9_gut_group*, *Desulfovibrio*, *Helicobacter*, *Anaerotruncus*, *Alloprevotella*, *Candidatus_Arthromitus*, *Lachnospiraceae_NK4A136_group*, and *Alistipes*. Furthermore, the LBT2 group was distinguished by a high prevalence of *Verrucomicrobiota*, *Cyanobacteria*, and *Bacteroidetes*, with the genera *Dubosiella*, *Akkermansia*, *Parasuttella*, *Lactobacillus, Bacteroides*, and *Paramuribaculum* being the most frequent in this ecological niche.

## 4 Discussion

In this study, we examined the regulatory role of LBT and the impact of Triton WR-1339 on hepatic lipid metabolism. The liver, the main organ responsible for lipid metabolism, can produce more lipids, especially triglycerides, as a result of consuming too many calories (Kathak et al., 2022). Abnormalities in lipoprotein synthesis may cause high levels of LDL and triglycerides in the bloodstream, which are significantly linked to the development of atherosclerosis (Singh, 2022). Triton WR-1339, a surfactant frequently employed in lipid metabolism research, can be delivered via intravenous, intramuscular, or intraperitoneal injections, leading to a substantial increase in blood cholesterol and triglyceride levels. This action may be facilitated by the suppression of lipoprotein lipase (Zarzecki et al., 2014; Khlifi et al., 2019; Majeed et al., 2021).

Animals receiving Triton WR-1339 had significantly higher levels of serum triglycerides, total cholesterol, low-density lipoprotein cholesterol, and very low-density lipoprotein cholesterol, but significantly lower levels of high-density lipoprotein cholesterol, which is consistent with the results are in agreement (Bouhlali et al., 2023). While the HDL-C level showed a progressive and significant increase with the increase in LBT concentration, the TG, TC, LDL-C, and VLDL-C levels in mouse blood showed a sequential and significant decrease, in line with prior findings that apigenin and eupatilin attenuate Triton WR-1339-evoked hyperlipidemia in mice (Kim et al., 2023; Hu et al., 2025). This suggests that Triton WR-1339-induced hyperlipidemia can be effectively reduced by LBT administration. Additionally, Triton WR-1339 alters the metabolism of hepatic lipids, resulting in fat buildup. The basic mechanisms may involve the inhibition of lipid-degrading enzymes and activation of signaling pathways associated with lipid synthesis (Palabiyik et al., 2023).

SREBP2 and HMGCR are essential regulatory proteins involved in cholesterol synthesis. The relationship between SREBP2 and HMGCR is essential for regulating cholesterol homeostasis in cells and reducing blood cholesterol levels (Ozkan-Nikitaras et al., 2024). Triton WR-1339 injection increases the expression levels of SREBP2 and HMGCR in murine livers, indicating that Triton WR-1339 boosts cholesterol synthesis in these organs, supporting previous findings (Tacherfiout et al., 2018). A dose-dependent reaction was observed in the expression of SREBP2 and HMGCR in the livers of hyperlipidemic mice after LBT administration (Figure 6). This finding demonstrates that LBT effectively inhibits cholesterol synthesis in the livers of hyperlipidemic mice. ABCA1 primarily facilitates the transfer of intracellular phospholipids and cholesterol to APOA1, a precursor of HDL, in reverse cholesterol transport, thereby initiating HDL synthesis (Chen et al., 2022). APOA1, primarily produced in the liver and small intestine, helps move excess cholesterol from peripheral tissues to the liver for processing and elimination. Reverse cholesterol transfer is a crucial process for controlling cholesterol levels in the body (Endres, 2021). LCAT, a plasma enzyme, catalyzes the transition of free cholesterol to cholesterol esters, thereby increasing HDL maturation through the esterification of free cholesterol (Kunnen and Van Eck, 2012; Hovingh et al., 2005). The findings of this study demonstrate that Triton WR-1339 induces the downregulation of ABCA1, APOA1, and LCAT expression, which is associated with a reduction in reverse cholesterol transport, consistent with prior studies (Zhang et al., 2024; Martínez-Beamonte et al., 2013; Zhao et al., 2015). Conversely, LBT increased the expression of these genes, promoting an increase in reverse cholesterol transport (Figure 5). LDLR, a membrane protein, is essential for the removal of LDL particles from plasma and functions as the principal transporter of cholesterol (Wang et al., 2016). Research indicates that Triton WR-1339 diminishes LDLR expression, resulting in increased LDL-C levels in the bloodstream (Askin and Umudum, 2024), whereas LBT augments LDLR expression, facilitating the clearance of LDL-C (Figures 4, 5), consistent with previously reported findings that (R)-(-)-carvone modulates LDLR expression (Abbas et al., 2020). Furthermore, APOB, the primary structural protein of VLDL and LDL, aids in the transport of cholesterol to peripheral tissues (Kurt et al., 2023). Research indicates that APOB expression is elevated in mice treated with Triton WR-1339, likely due to the activation of the PI3K/AKT/GSK signal mechanism. As a result of this activation, transcription factors linked to the APOB gene become more active, which in turn increases its expression (Han et al., 2009). This study demonstrated that APOB gene expression was increased in the livers of mice after administration of Triton WR-1339; however, pretreatment with LBT led to a reduction in APOB gene expression in correlation with increasing doses of LBT (Figure 5). This corroborates the notion that LBT diminishes APOB gene expression in murine livers, consistent with prior studies indicating that ginseng extract produces a similar effect in hyperlipidemic mouse livers (Jin and Wang, 2024). We will subsequently examine the molecular pathways by which LBT affects cholesterol production and reverse transport using cellular models.

Previous studies have established a significant correlation between Triton WR-1339-induced hyperlipidemia and inflammatory diseases. Triton WR-1339 stimulates the MAPK signaling pathway, resulting in cellular growth and generation of inflammatory mediators (Xiao et al., 2023). Moreover, it stimulates the NF-κB pathway, resulting in the release of inflammatory cytokines, including TNF-α and IL-6, thereby intensifying the inflammatory response (De la Rosa-García et al., 2022; Palumbo et al., 2024). By disrupting the TLR4/NF-κB signaling pathway, LBT notably reduced inflammation in the colons of hyperlipidemic mice. However, this study did not provide protein-level or phosphorylation activity data for the TLR4/NF-κB pathway; therefore, the regulatory effect of LBT on this pathway awaits confirmation by western blot or immunofluorescence assays. Furthermore, LBT strengthened mucosal immune function and anti-inflammatory capabilities in the colons of hyperlipidemic mice by enhancing the availability of anti-inflammatory cytokines, including IL-4, IL-10, and secretory immunoglobulin A (Figures 6, 7). Nevertheless, additional research is required to gain a comprehensive understanding of the intricate pathways that contribute to the effects of LBT on the body. Furthermore, we found that Triton WR-1339 caused a Th1/Th2 imbalance in the colon of mice, leading to an increased Th1/Th2 ratio, whereas LBT decreased this ratio (Figure 6). When IFN-γ induces macrophages to adopt the M1 phenotype, which results in the release of IFN-γ and IL-6, they become more susceptible to inflammation (Arabpour et al., 2021). Chronic inflammation induced by Th1 cells results in deficiencies in tissue healing (Soliman and Barreda, 2022). Nonetheless, the signaling pathway through which LBT modulates the Th1/Th2 equilibrium requires further examination.

Chronic disorders, such as obesity, diabetes, and atherosclerosis, are influenced by the gut microbiota, which also controls the host’s metabolic processes (Nisar et al., 2021). Various external factors affect the composition of the gut microbial population, including geographical location, age, genetic predisposition, dietary selection, and the use of prebiotics and antibiotics (Kennelly et al., 2018). The hyperlipidemic group of Triton WR-1339 may compromise the lipid bilayer integrity of intestinal epithelial cell membranes, triggering apoptosis (Jia et al., 2021), diminishing mucin (MUC2) secretion, impairing the intestinal physical barrier, and facilitating pathogen colonization (Luangphiphat et al., 2024). The examination of α diversity in this study revealed no significant changes, which may be attributable to the restricted sample size. The assessment of β-diversity indicated that Triton WR-1339 and LBT resulted in unique gut microbiota compositions in mice relative to the control group. Subsequent examination of the fecal microbiota composition across the three mouse groups indicated significantly higher amounts of *Deferribacterota*, *Desulfobacterota*, *Campylobacterota*, and *Patescibacteria* in the model group than in the control group. The injection of LBT resulted in a reduction in the abundance of *Deferribacterota*, *Desulfobacterota*, *Campylobacterota*, and *Firmicutes*, while simultaneously increasing the levels of *Verrucomicrobiota* and *Cyanobacteria*. Several species from the phylum *Verrucomicrobiota* are recognized as probiotics that help alleviate symptoms associated with obesity-related diseases. Conversely, several *Firmicutes* species are positively correlated with the incidence of these disorders (Gangzheng et al., 2023). *Verrucomicrobiota*, *Cyanobacteria*, and *Bacteroidetes* were significantly more abundant in the gut of mice in the LBT group, whereas *Desulfobacterota*, *Firmicutes*, *Campylobacterota*, and *Desulfobacterota* were the main phyla in the model group. *Verrucomicrobiota* abundance was negatively correlated with TC, TG, and LDL-C levels, but positively correlated with HDL-C, IL-10, and secretory immunoglobulin A levels. *Cyanobacteria* had a favorable correlation with IL-10 and SIgA levels. Our findings indicate that a reduction in *Verrucomicrobiota* abundance may result in an increase in *Campylobacterota* abundance, suggesting a potential competing or antagonistic interaction between these two phyla in sustaining intestinal homeostasis (Babacan Yildiz et al., 2023). The reduction of Verrucomicrobiota may instigate metabolic problems, including obesity and type 2 diabetes, whereas the proliferation of Desulfobacterota could intensify intestinal inflammation via hydrogen sulfide toxicity (Lu et al., 2024). Under anaerobic conditions, *Deferribacterota* and *Desulfobacterota* may interact via iron and sulfur metabolism, with *Deferribacterota* promoting the proliferation of *Desulfobacterota* through iron reduction, whereas *Desulfobacterota* modify the ecological niche of *Deferribacterota* through sulfur metabolism (Li et al., 2022). The heatmap analysis of microbial phyla revealed a positive correlation between *Verrucomicrobiota* and *Cyanobacteria* and a negative correlation between *Verrucomicrobiota* and *Deferribacterota*, *Desulfobacterota*, and *Campylobacterota*. *Deferribacterota*, *Desulfobacterota*, and *Campylobacterota* exhibited positive relationships with each other, while demonstrating negative associations with *Cyanobacteria*. An increased ratio of Firmicutes to Bacteroidetes (F/B) has been observed in patients with hyperlipidemia (Wang et al., 2024), whereas a decrease in this ratio has been associated with several bioactive chemicals with hypolipidemic properties. Glycosaminoglycans, for instance, can aid in reestablishing gut microbiota balance by reducing the Firmicutes/Bacteroidetes ratio (Kong et al., 2021; Ren et al., 2023). Similarly, by reducing the F/B ratio, *Lactobacillus acidophilus* can treat the gut flora imbalance caused by a high-fat diet (Kang et al., 2022; Ren et al., 2023). We observed that the F/B ratio increased 1.15 times when Triton WR-1339 was administered; however, LBT effectively eliminated the elevation caused by Triton WR-1339.

Based on our findings, several genera, such as *Odoribacter*, *Oscillibacter*, *Desulfovibrio*, *Colidextribacter*, *Anaerotruncus*, *Helicobacter*, *Candidatus_Arthromitus*, *Acetatifactor*, and *Alloprevotella*, showed enhanced proliferation after Triton WR-1339 treatment, whereas *Ruminococcus*, *Ligilactobacillus*, *Erysipelatoclostridium*, *Parasutterella*, *Clostridium*, and *Prevotellaceae_UCG-001* had their growth inhibited (see Figure 10). Linear Discriminant Analysis Effect Size to Taxonomy Indicator Bubnle Plot analysis identified *Acetatifactor*, *Oscillibacte*r, *Odoribacter*, *Candidatus_Arthromitus*, *Desulfovibrio*, *Anaerotruncus*, *Helicobacter*, and *Alloprevotella* in the intestines of model group mice (Figure 10). Correlation analysis revealed that TC, TG, LDL-C, VLDL-C, ALT, AST, IL-6, and TNF-α showed positive relationships with *Acetatifactor*, *Desulfovibrio*, *Odoribacter*, *Candidatus_Arthromitu*s, and *Helicobacter*, while HDL-C, IL-4, and SIgA showed negative correlations with these bacteria. *Oscillibacter* abundance was positively correlated with TC, TG, LDL-C, VLDL-C, ALT, AST, and TNF-α, and negatively correlated with HDL-C, IL-10, and SIgA. *Desulfovibrio*, acknowledged as a prominent sulfate-reducing bacterium (SRB), is vital to the gut environment. Research indicates that it may significantly impact the onset of hyperlipidemia by influencing inflammatory responses and lipid storage (Pi et al., 2024). In inflammatory disorders, such as inflammatory bowel disease, *Desulfovibrio* prevalence generally increases and correlates positively with endotoxin (LPS) levels (Zhang et al., 2023a). TC and LDL-C, two indicators of host lipid metabolism, are often positively correlated with *Odoribacter* abundance in hyperlipidemia models (Yun et al., 2020). The increase in *Odoribacter* abundance may be related to increased intestinal permeability and decreased flora diversity (Xiao et al., 2023). Moreover, *Odoribacter* and *Desulfovibrio* may demonstrate metabolic complementarity by utilizing shared carbon sources, such as sulfate, which suppresses the proliferation of other symbiotic bacteria, including *Lactobacillus* (Koh et al., 2016). *Candidatus_Arthromitus* is thought to be intricately connected to host metabolic diseases, potentially affecting host lipid metabolism by modulating adipogenesis and the production of diabetes-associated enzymes (Lin et al., 2016). The metabolism of gastrointestinal microbiota may be altered by a decrease in abundance, which could potentially exacerbate obesity (Kumar et al., 2021). *Helicobacter pylori* (H. pylori) infection is significantly correlated with changes in host lipid metabolism. This may lower HDL-C, raise LDL and total cholesterol levels, and activate proinflammatory cytokine production, thereby altering adipocyte function (Martín-Núez et al., 2020; Temesgen et al., 2022; Xiao et al., 2024). *Acetatifactor* may affect the function and composition of the microbial community through interactions with other microorganisms, potentially influencing the host’s metabolic health and immunological response (Zhang et al., 2023b). Triton WR-1339 enhanced *the abundance of Acetatifactor*, whereas LBT diminished it. LBT treatment markedly increased the prevalence of *Akkermansia*, *Ruminococcus, Dubosiella*, *Erysipelatoclostridium*, *Parasutterella*, *Prevotellaceae_UCG-001*, *Lactobacillus*, *Paramuribaculum*, and *Bacteroides* in the intestines of hyperlipidemic mice, while concurrently decreasing the prevalence of *Odoribacter*, *Desulfovibrio*, *Oscillibacter*, *Colidextribacter*, *Anaerotruncus*, *H. pylori*. The existence of several genera, such as *Dubosiella*, *Akkermansia*, *Parasutterella*, *Lactobacillus*, *Bacteroides*, and *Paramuribaculum*, distinguishes the LBT2 group. Conversely, atorvastatin suppresses the proliferation of *Akkermansia muciniphila* and *Lactobacillus* in the intestines of high-fat-diet mice and patients with coronary artery disease (Wang et al., 2021; Cheng et al., 2021).

Subsequent studies have revealed that *Dubosiella* is negatively associated with TC, TG, LDL-C, ALT, and AST but positively correlated with IL-10 and SIgA, which is consistent with the findings of Cheng et al. (Cheng et al., 2022). Zhao et al. revealed that intervention with *Dubosiella newyorkensis* not only significantly alleviated dyslipidemia but also induced a 43 % increase in Lactobacillus abundance, with this synergistic effect being directly mediated by acetate secreted by the bacterium (Zhao et al., 2025). These findings suggest that *Dubosiella* may help prevent liver disease by enhancing liver metabolism and reducing the incidence of fatty liver, possibly due to its ability to regulate inflammation and metabolic responses (Liu et al., 2023). *Akkermansia* plays a crucial role in addressing metabolic disorders, such as obesity and hypertriglyceridemia, owing to its adverse associations with total cholesterol, triglycerides, low-density lipoprotein cholesterol, alanine aminotransferase, and aspartate aminotransferase, and its favorable associations with high-density lipoprotein cholesterol, interleukin-10, and secretory immunoglobulin A (Zhou et al., 2021; Yavorov-Dayliev et al., 2023; Gao et al., 2025). Studies have shown that acetate produced by *A. muciniphila* exerts an anti-ferroptotic effect against MAFLD by activating the hepatic AMPK/SIRT1/PGC-1α axis and suppressing PUFA synthesis (Zhuge et al., 2025). Concurrently, the bacterium attenuates lipid uptake in mouse and human jejunal epithelial cells via AMPK phosphorylation (Ma et al., 2025), while its outer membrane proteins further potentiate AMPK signaling through TLR2, inhibit NF-κB activity, reduce inflammation, and reinforce the intestinal barrier integrity of Caco-2 monolayers (Shi et al., 2022). *Parasutterella* was negatively correlated with total cholesterol, total fat, low-density lipoprotein cholesterol, very low-density lipoprotein cholesterol, alanine aminotransferase, and tumor necrosis factor-alpha, and positively correlated with interleukin-10 and serum immunoglobulin A. Metabolic issues associated with high-fat diets have been linked to a decline in the abundance of *Parasutterella*. (Mu et al., 2020; Ju et al., 2019). *Parasutterella*-derived metabolites markedly reshaped the profiles of aromatic amino acids, bilirubin, purines, and bile acid derivatives. Concomitantly, the expression of ileal bile acid transporter genes and hepatic bile acid synthetic genes is correspondingly up- or downregulated, implying that *Parasutterella* plays a pivotal role in maintaining bile acid homeostasis and modulating cholesterol metabolism (Ju et al., 2019). *Lactobacillus* has good probiotic potential and is beneficial for lipid reduction in animals (Yi et al., 2020). Recent studies have shown that *Lactobacillus fermentum*, *Lactobacillus plantarum*, and *Lactobacillus reuteri 8513* can remodel cellular energy homeostasis by phosphorylating 5’ adenosine monophosphate-activated protein kinase (AMPK), thereby alleviating hyperlipidemia and hepatic steatosis in aged rats (Lew et al., 2020). Short-chain fatty acids (SCFAs) secreted by *Lactobacillus fermentum E15* activate GPR43 and markedly attenuate hyperlipidemia induced by a hypercholesterolemic diet (Chen et al., 2025). *Bacteroides* are well-known to play a large role in maintaining eubiosis in the intestine. This is due to the multiple beneficial interactions within the gut microbiota (Shin et al., 2024; Shin et al., 2024). For example, Xu et al. demonstrated that *B. vulgatus* Bv46 treatment restored the gut microbiota structure, reshaped the metabolic profile, and expanded the pools of bile acids (BAs) and short-chain fatty acids (SCFAs), thereby accelerating energy expenditure, alleviating systemic inflammation, promoting cholesterol excretion, and improving lipid homeostasis in rats fed a high-fat diet (Xu et al., 2023). Li et al. demonstrated that *Bacteroides thetaiotaomicron* reshapes the gut microbiota, significantly lowering the Firmicutes/Bacteroidetes ratio in HFD-fed mice, while concurrently elevating gut–liver folate levels and hepatic metabolites to ameliorate metabolic dysfunction (Li et al., 2024). Research has demonstrated that *Akkermansia muciniphila*, which serves as a key degrader of the mucus layer, can decompose mucin, leading to the production of acetic acid, which represents more than 70 % of its metabolites. This process generates a carbon source for *Dubosiella*, thereby facilitating the synthesis of butyric acid (Li S. et al., 2025). Additionally, *Paramuribaculum* transforms conjugated bile acids into unconjugated bile acids through the expression of β-glucuronidase, which activates FXR receptors and inhibits sulfatase activity in *Bacteroides* (Chen et al., 2023; Felicianna et al., 2025). The D-lactate that *Lactobacillus* secretes can be converted into propionic acid by the LDH isomerase produced by *Parasuttella*, while also suppressing the propionic acid kinase activity of *Dubosiella* (Pessione, 2012). Moreover, reuterin synthesized by *Lactobacillus reuteri* can upregulate genes associated with the oxidative stress response in *Escherichia coli*, thereby bolstering its antimicrobial capacity and forming a symbiotic defense alliance (Li J. et al., 2025). Consequently, the role of LBT in regulating lipid metabolism disorders and inflammation may be linked to its interaction with microbial networks. Our team will delve into the mechanisms by which LBT influences the microbial network, bile acid metabolism, short-chain fatty acids, and other interrelationships in hyperlipidemic mice.

## 5 Conclusion

In conclusion, LBT can reduce blood lipid levels, mainly by inhibiting endogenous cholesterol production and promoting the reverse transport of cholesterol in the livers of hyperlipidemic mice. Specifically, LBT exerts anti-inflammatory effects by interacting with the TLR4/NF-κB pathway. Additionally, it modifies the Th1/Th2 immunological balance, ensuring a healthy immune response. Moreover, LBT has demonstrated the ability to reestablish the ecological equilibrium of gut microbiota in high-fat mice, promoting the proliferation of advantageous taxa, including *Dubosiella*, *Akkermansia*, *Parasuttella*, *Lactobacillus*, *Bacteroides*, and *Paramuribaculum*. This improvement supports the gut microbiota network, metabolism-microbiota axis, and immune-microbiota interactions, ultimately favoring the growth of beneficial bacterial species. The conclusions of the present study apply only to female KM mice; extrapolation to male or other strains should be approached with caution.

## Data Availability

The original contributions presented in the study are publicly available. This data can be found in here: 10.6084/m9.figshare.29821130.
